# Community-based rehabilitation intervention for people with schizophrenia in Ethiopia (RISE): a 12 month mixed methods pilot study

**DOI:** 10.1186/s12888-018-1818-4

**Published:** 2018-08-03

**Authors:** Laura Asher, Charlotte Hanlon, Rahel Birhane, Alehegn Habtamu, Julian Eaton, Helen A. Weiss, Vikram Patel, Abebaw Fekadu, Mary De Silva

**Affiliations:** 1 Division of Epidemiology and Public Health, School of Medicine, University of Nottingham, Nottingham, NG5 1PB UK; 2 Centre for Global Mental Health, London School of Hygiene and Tropical Medicine, London, UK; 3 Department of Psychiatry, College of Health Sciences, Addis Ababa University, Addis Ababa, Ethiopia; 4 Centre for Global Mental Health, Health Services and Population Research Department, Institute of Psychiatry, Psychology and Neuroscience, King’s College London, London, UK; 5 CBM International, Cambridge, UK; 6 MRC Tropical Epidemiology Group, Department of Infectious Disease Epidemiology, London School of Hygiene and Tropical Medicine, London, UK; 7 Department of Global Health and Social Medicine, Harvard Medical School, Boston, USA; 8 Centre for Innovative Drug Development and Therapeutic Trials for Africa (CDT-Africa), College of Health Sciences, Addis Ababa University, Addis Ababa, Ethiopia; 9 Centre for Affective Disorders, Department of Psychological Medicine, Institute of Psychiatry, Psychology and Neuroscience, King’s College London, London, UK; 10 Wellcome Trust, London, UK

**Keywords:** Schizophrenia, Psychotic disorders, Community health services, Psychiatric rehabilitation, Feasibility studies, Ethiopia, Developing countries, Community based rehabilitation, Community based inclusive development

## Abstract

**Background:**

Community-based rehabilitation (CBR), or community-based inclusive development, is an approach to address the complex health, social and economic needs of people with schizophrenia in low and middle-income countries. Formative work was undertaken previously to design a culturally appropriate CBR intervention for people with schizophrenia in Ethiopia. The current study explored the acceptability and feasibility of CBR in practice, as well as how CBR may improve functioning among people with schizophrenia.

**Methods:**

This mixed methods pilot study took place in rural Ethiopia between December 2014 and December 2015. Ten people with schizophrenia who were unresponsive to treatment with medication alone, and their caregivers, participated in CBR. CBR was led by lay workers with five weeks training and involved home visits (education, family intervention and support returning to work) and community mobilisation. Theory of change was used to guide the pilot evaluation. Qualitative and quantitative data were collected at baseline, six months and 12 months. Forty in-depth interviews and two focus group discussions were conducted with 31 individuals comprising people with schizophrenia, caregivers, CBR workers, supervisors, health officers and community members.

**Results:**

The RISE CBR intervention may have a positive impact on functioning through the pathways of enhanced family support, improved access to health care, increased income and improved self-esteem. CBR was acceptable to CBR workers, community leaders and health officers. Some CBR workers found it challenging to accept the choices of people with schizophrenia. These concerns were felt to be resolvable with supplementary training for CBR workers. The intervention was feasible but further evaluation is needed on a larger scale.

**Conclusion:**

In low and middle-income countries, CBR may be an acceptable and feasible adjuvant approach to facility-based care for people with schizophrenia. However, contextual factors, including poverty and inaccessible anti-psychotic medication, remain substantial challenges. There were indications that CBR can impact on functioning but the RISE trial will determine effectiveness.

**Electronic supplementary material:**

The online version of this article (10.1186/s12888-018-1818-4) contains supplementary material, which is available to authorized users.

## Background

Schizophrenia can have profound and enduring effects on individuals, families and communities. Many people with schizophrenia have difficulties with work and social participation [1, 2] and the impacts may be worse in low and middle-income countries (LMIC) such as Ethiopia. The prevalence of violent victimization is significantly higher in people with severe mental illness compared to the general population of rural Ethiopia (60.7% vs. 41.5%) [3] and some individuals may experience human rights abuses such as physical restraint [4]. Furthermore, mortality rates in this setting are three times greater than the general population [5]. In LMIC high levels of disability amongst people with schizophrenia arise from a combination of (i) illness factors such as low motivation, cognition, communication problems and poor physical health and (ii) societal factors including stigma, poverty and poor access to treatment (only 10% of people with schizophrenia access biomedical care in rural Ethiopia) [6, 7]. One reason for the treatment gap is the scarcity of mental health specialists. There are currently only around 70 psychiatrists working in Ethiopia (approximately 0.7/ 1,000,000 population) [8].

The same considerations that make schizophrenia a priority disorder for global health action also render it uniquely challenging to address. Novel and holistic approaches for supporting people with schizophrenia are needed, which harness the powerful influence of the community for the benefit of individuals and utilise a non-specialist workforce. Community-based rehabilitation (CBR), which combines support for individuals and families with community mobilisation, may meet this need. There is a well-established global network of CBR programmes, which have traditionally supported people with physical disabilities in the domains of health, social life, livelihoods, education and empowerment [9, 10]. CBR is increasingly known as community-based inclusive development, reflecting a growing emphasis on the goal to be achieved rather than the process; however, the term CBR will be used in this paper. CBR programmes vary and delivery personnel may include paid non-specialists, volunteers, physiotherapists, community nurses, and teachers [9]. For example, the Rehabilitation and Prevention Initiative against Disability (RAPID) programme in Adama, Ethiopia, enabled local field workers, teachers and parent groups to support the physical rehabilitation and social inclusion of children with disabilities (https://www.cbm.org/Rehabilitation-and-education-in-Ethiopia-488115.php). More recently CBR has been tailored for people with schizophrenia in LMIC [11].

In the last eight years mental health has been integrated into primary care in several pilot sites in Ethiopia, guided by the World Health Organisation’s (WHO) Mental Health Gap Action Programme (mhGAP) [12]. These endeavours have increased the availability of antipsychotic medication in rural areas. However mental health service users, care providers and community leaders in rural Ethiopia have also called for services that enhance access to biomedical care, and equip families to provide emotional and practical support and support livelihoods [13]. CBR is recommended in the Disease Control Priorities-3 as an appropriate intervention for schizophrenia in LMIC [14], and has the potential to meet the expressed needs of local stakeholders in rural Ethiopia as well as attending to the shortage of mental health professionals in these settings. Community-based psychosocial interventions may improve functioning and symptoms in people with schizophrenia in middle-income countries [15]. However much of the available evidence relates to intensive programmes involving care delivery by mental health specialists, and few incorporate a community mobilisation element. To our knowledge there have been no randomised controlled trials (RCT), or studies investigating the feasibility or acceptability, of CBR for schizophrenia in any low-income country setting. A systematic review concluded that psychosocial interventions in middle-income countries are acceptable to participants but that evidence on feasibility is limited. Furthermore most interventions were set in outpatient clinics in urban areas, were delivered by mental health specialists and did not reflect the CBR model [16]. An RCT of community-based care for schizophrenia delivered by non-specialists conducted in a middle income country (India) had a more powerful effect in areas with the fewest resources [11]. The intervention had good acceptability and feasibility following adaptations [17].

As part of the Rehabilitation Intervention for people with Schizophrenia in Ethiopia (RISE) project, in-depth formative work was previously undertaken to design an acceptable, feasible and culturally appropriate intervention for the Ethiopian context [18]. The current paper describes a subsequent 12-month pilot of this CBR intervention for people with schizophrenia in rural Ethiopia to assess acceptability and feasibility in practice. The study aimed to answer three research questions:

1. Is the RISE CBR intervention acceptable?
2. Is the RISE CBR intervention feasible?
3. Can the RISE CBR intervention produce an impact and if so, how?

An additional objective was to test procedures for a subsequent definitive trial (NCT02160249) [19]. The paper details the adjustments made to the intervention in preparation for the RISE trial.

## Methods

### Study design

We conducted a mixed methods pilot study involving quantitative, qualitative and process data collection [20]. This study represents the piloting/feasibility phase of the Medical Research Council framework for the development and evaluation of complex interventions [21]. The core objectives of this stage are to pilot procedures for acceptability and feasibility and estimate key parameters such as retention rates. The MRC guidance recommends both qualitative and quantitative methods are used [21]. Further MRC guidance promotes the use of process evaluation as part of feasibility testing, including consideration of intervention fidelity, mechanisms of impact and context [22]. Theory of change is a theory driven approach to operationalise the MRC framework that has been used through all stages of RISE intervention development, piloting and evaluation [23].

**Fig. 1:**
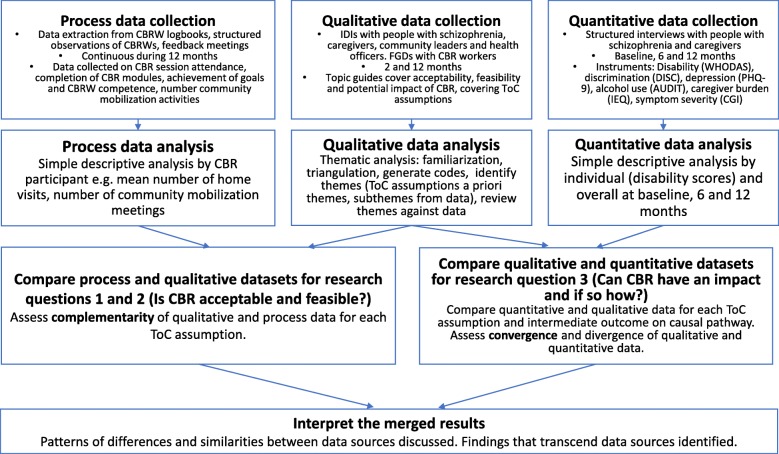
Parallel convergent mixed methods model

CBR was delivered over a 12 month period. Process data were collected continuously throughout the 12 months to answer questions 1 and 2 (acceptability and feasibility of CBR). Quantitative data were collected at baseline, 6 months and 12 months to answer research question 3 (potential impact of CBR). Qualitative data were collected at 2 months and 12 months and were used to answer all three research questions. A parallel convergent mixed methods analytical design was used (see Fig. 1) [20]. See Additional file 1 for the piloting of trial procedures.

### Setting

The study was conducted in Sodo district in the Gurage zone of the Southern Nations, Nationalities and Peoples’ Region of Ethiopia. The district is approximately 100 km from Addis Ababa. The majority of the population of Sodo live in difficult to reach rural areas. Most of the population are subsistence farmers and live in one-room mud and straw houses. Around 55% of the population is literate. The majority of the population are Orthodox Christian. Most people are members of an *edir* (traditional support association) group [18]. Traditional healing and holy water are commonly utilised for mental health problems. Holy water, which is believed to have curative properties, is accessed at sites associated with the Ethiopian Orthodox Church [24].

Sodo district is the setting for the Ethiopian arm of the PRogramme for Improving Mental healthcarE (PRIME) research consortium. As part of PRIME, primary care staff in Sodo were trained to assess people with schizophrenia and offer psychotropic medication, psychoeducation and follow-up using mhGAP guidance [12]. This model of care is part of the 2012 Ethiopian National Mental Health Strategy [8]. Care costs were largely out-of-pocket with a fee waiver available for the poorest. PRIME identified people with schizophrenia in Sodo using the key informant method and, from December 2014, began following up those accessing facility-based care in a 12-month cohort study.

### Participants

This pilot study was conducted between December 2014 and December 2015. Participants were recruited at the baseline of the PRIME cohort study. The participants were ten people with schizophrenia and their families living in four sub-districts. The intervention coordinator assessed consecutive PRIME cohort recruits for RISE pilot eligibility by reviewing PRIME baseline data. There were no specific exclusion criteria. Participants meeting all of the following criteria were included:

1. Participant in PRIME cohort study
2. No immediate plans to leave the subdistrict
3. Has a primary caregiver who is willing to participate
4. Age ≥ 18 years
5. Diagnosis of schizophrenia spectrum disorder (schizophrenia, schizoaffective disorder or schizophreniform disorder) using DSM-IV criteria (assessed using the Operational Criteria for Research (OPCRIT)) and
6. Evidence of enduring or disabling illness demonstrated by ≥1 of:
  (i) Brief Psychiatric Rating Scale – Expanded version (BPRS-E) score ≥ 52 [25]
  (ii) 36-item WHODAS 2.0 score ≥ 35 [26]
  (iii) Continuous illness over the preceding six months, assessed by Life Chart Schedule [27]
  (iv) Symptomatic in ≥3 of the last six months, assessed by Longitudinal Interval Follow-up Evaluation [28] or
  (v) Clinical Global Impression (CGI) score ≥ 4 (at least moderately ill) [29].

### CBR intervention

All participants received CBR in addition to the PRIME intervention at the health centre. CBR was delivered by 10 CBR workers (CBRWs), who were lay persons recruited from the local area with a minimum of eight years of primary and two years of secondary education but no prior mental health experience. They received five weeks training in CBR delivery, guided by a 200-page RISE manual translated into Amharic by Ethiopian psychiatrists [30]. The RISE training programme and manual were designed to address the CBR worker competencies (Additional file 2). Training was equally split between classroom teaching and fieldwork. Classroom-based training was delivered in Amharic by psychiatrists and RAPID CBR coordinators and included role-plays, whole group discussions, viewing communication skills videos, and quizzes. Fieldwork included shadowing RAPID CBR workers, observing psychiatric nurse clinics and making home visits to persons with schizophrenia.

Two or three CBRWs were based in each sub-district and each CBRW supported one person with schizophrenia and their caregiver over a 12-month period. The low workload was intentional to ensure all CBRWs could be trained for the RISE trial. The CBR intervention is described in detail elsewhere [18]. In brief it comprised: (i) **Home visits** by CBRWs, initially weekly reducing to two-weekly then monthly, lasting 30–90 min. Topics included psychoeducation, adherence support, family intervention, crisis management, support returning to work and social activities, and dealing with stigma and stress. CBRWs were trained in basic counselling and problem-solving techniques (see RISE Manual Chapters 10 and 11 [30]); (ii) **Community mobilisation** including community awareness-raising and targeted mobilisation of financial or practical support depending on individual need; and (iii) **family support groups**. The intervention was recovery-oriented, emphasising hope, human rights, and the participants’ own goals. Intervention delivery was guided by the RISE manual. There were four core goals relating to understanding of schizophrenia, access to healthcare, human rights and crisis management. CBR participants were supported to select optional goals from a pre-defined list covering functioning, symptoms and stigma (Additional file 3). No financial support or medication was provided by CBRWs. Two supervisors and a project coordinator oversaw the frequency, content and quality of CBR. Supervision for CBRWs included bi-monthly observed sessions, and monthly group supervision and individual supervision. Top-up training was given as required. CBRWs referred participants to the health centre, in addition to regular appointments, if suicide intent, relapse, physical illness or medication side-effects were identified. Primary care staff did not directly contribute to CBR.

### Theory of change

**Fig. 2:**
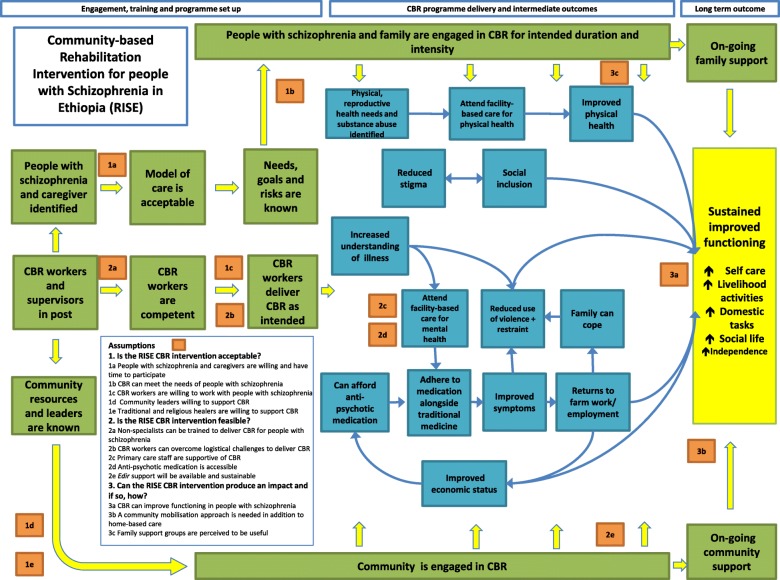
Pre-pilot theory of change

Theory of change was used to guide the pilot evaluation. Theory of change has been defined as, “an approach which describes how a programme brings about specific long-term outcomes through a logical sequence of intermediate outcomes” [31]. Figure 2 presents the RISE theory of change map that was produced in the intervention development phase. ‘Sustained improved functioning’ was the long-term outcome (yellow box). Intermediate outcomes related to programme delivery (green boxes) and causal pathways to improved functioning (blue boxes). Thirteen assumptions were identified (oranges boxes), representing what needs to be in place to proceed through intermediate outcomes to achieve the final outcome. The pilot evaluation was designed to test the assumptions, grouped into the three research questions.

### Data collection and analysis

#### Qualitative

The qualitative component used a thematic analysis from a realist perspective, meaning that whilst all speech was not taken purely at face value, sub-themes were generally identified by examining the surface (semantic) meanings of the data [32]. The findings were then contextualised by considering the social and economic setting in which experiences took place. Qualitative data were collected at two and 12 months. A total of 40 in-depth interviews (IDIs) were conducted with 21 individuals comprising people with schizophrenia, caregivers, supervisors, primary care staff and community members (Table 1). Of the 20 people with schizophrenia and caregivers who participated in CBR, 15 were interviewed at both two months and endline. Two people with schizophrenia, both with co-morbid intellectual disability, were assessed not to have the cognitive capacity to participate in IDIs. Two caregivers and a man with schizophrenia declined to be interviewed at two months and three caregivers declined at endline. Focus group discussions (FGDs) were held with all CBRWs at each time point. IDIs were held with the two CBR supervisors. The two health officers who provided facility-based mental health care were included. Three community members engaged in CBR (a priest, a health extension worker and a businessman) were included to triangulate different perspectives on CBR. The topic guides addressed the research questions, namely the acceptability, feasibility and perceived impact of CBR, with specific questions guided by the assumptions (see Additional file 4).

**Table 1:** IDI and FGD participants

Participant type	2 months		12 months	
	IDIs	FGDs	IDIs	FGDs
Men with schizophrenia	4	0	5	0
Women with schizophrenia	3	0	3	0
Male caregivers	1	0	1	0
Female caregivers	7	0	6	0
CBR supervisors	2	0	2	0
CBRWs	1	2 (n = 10)	0	2 (n = 10)
Health officers	2	0	0	0
Community members	0	0	3	0
Total	20	2 (n = 10)	20	2 (*n* = 10)

Participants received modest remuneration for their time and transportation. IDIs and FGDs were conducted in Amharic at participants’ homes or a research office by an Ethiopian research assistant with a social work Masters degree (AH) who had experience in qualitative work with people with schizophrenia [33] . IDIs lasted a mean of 46 min. Audio-recordings were transcribed in Amharic and then translated into English. The first two interviews were rapidly transcribed, translated and reviewed, prior to conducting further interviews. Feedback was given to AH on interviewing technique and appropriate use of the topic guide. Debriefing between AH and LA continued at regular intervals throughout data collection. As qualitative data collection progressed, questions were added to explore issues arising from the initial IDIs and from ongoing process data collection. LA clarified with AH ambiguous translations or cultural references. To triangulate the data linked transcripts from the person with schizophrenia, caregiver, CBRW and community member were read in batches. The aim was to construct an understanding of events and experiences for each participant, whilst acknowledging that no single version of events could be considered the ‘truth’. Note was made of changes and contradictions in experiences and opinions over time and between linked participants. Thematic analysis was conducted, using NVivo for Mac software to manage the data [32]. After independently coding two transcripts LA and CH discussed differences and made adjustments to the coding scheme. LA indexed all manuscripts using the final coding scheme, then collated the codes into themes. The coding framework was based around a priori high-level themes aligned with the assumptions, for example ‘Assumption 1a: People with schizophrenia and caregivers are willing and have time to participate in CBR’. An inductive approach was employed to identify sub-themes, in this case specific reasons for participation and non-participation. Attention was paid to associations with participant characteristics. Deviant cases were identified and incorporated into the framework. A selection of the full transcripts were reread to confirm that the final thematic framework adequately reflected the data collected. At all stages in the data collection and analysis process detailed notes were kept by LA on methodological decisions and rationale.

#### Quantitative

Quantitative data were collected from CBR participants at baseline, six and 12 months as part of the PRIME psychosis cohort study. Participants received travel expenses. Trained lay data collectors interviewed people with schizophrenia using a questionnaire comprising: the Discrimination and Stigma Scale-12 (DISC-12) subscale 1 [34], the Alcohol Use Disorders Identification Test (AUDIT) [35] and the Patient Health Questionnaire-9 (PHQ-9) [36] to measure depression. Data collectors administered a questionnaire to caregivers comprising of the Involvement Evaluation Questionnaire (IEQ) to measure caregiver burden [37] and the proxy-reported 36-item World Health Organisation Disability Assessment Schedule (WHODAS) 2.0 [26] to measure disability amongst people with schizophrenia. A psychiatric nurse rated the Clinical Global Impression [29]. All instruments have been translated into Amharic and validated or adapted for use in Ethiopia [26, 36]. Further details on the instruments can be found in Additional file 5. Trained research assistants verified questionnaires immediately after data collection, and any missing items or discrepancies were clarified with the participants. Data were double entered onto Epidata databases and descriptive summaries were prepared using Stata Version 12 [18].

#### Process

Process data were compiled to determine the fidelity and quality of CBR delivery including (i) two-weekly meetings with CBRWs to discuss participants’ progress and barriers faced, (ii) quantitative implementation data extracted from CBRW logbooks and (iii) structured observations of CBRWs. Descriptive summaries of process data were prepared using Stata Version 12.

#### Qualitative, quantitative and process data synthesis

The quantitative, qualitative and process data were analysed separately (see fig. 1). We assessed complementarity between process and qualitative data in relation to research questions 1 and 2 (acceptability and feasibility of CBR). We assessed convergence between qualitative and quantitative findings in relation to research question 3 (potential impact of CBR). For each theory of change assumption in turn, the results from each relevant data source were viewed side-by-side to identify areas of convergence and divergence. For assumption 3a (CBR can improve functioning) the individual quantitative outcome scores were compared to accounts from IDIs. An initial analysis was conducted after six months and a final analysis after 12 months. For assumptions that were not initially met, iterative adjustments were made to ensure that the final intervention package was acceptable, feasible, and likely to improve functioning.

## Results

### Overview

The CBR participants comprised five men and five women with schizophrenia, aged between 19 and 60 years. All female participants were illiterate whilst male participants had between five and eight years of school education. All caregivers were female (wives, mothers, sisters and a daughter) except one male benefactor who was not a relative. Two did not have active caregivers. The mean duration of illness was 10 years (range 1 to 30 years). At recruitment into the pilot, half of participants were treatment naïve and only one participant was taking medication. Linked identification numbers are used for people with schizophrenia, caregivers, CBRWs and community members; ‘B’ and ‘E’ indicate baseline and endline interviews. Additional file 6 contains a summary of the assumptions, findings and adjustments; Additional file 7 contains supporting qualitative data.

### Is the RISE CBR intervention acceptable?

#### Assumption 1a: People with schizophrenia and caregivers are willing and have time to participate

Participants received a mean of 21 home visits (range 17–27 visits) (Table 2), matching the anticipated total. CBR participants generally welcomed the service, and most described a good relationship with the CBRW. Most CBR participants said they could rely on the CBRW. For one caregiver, the fact the CBRW was from the local area was important. CBRWs felt that a trusting relationship was essential for positive outcomes. The majority of CBR participants were happy with regular home visits, often disregarding the time cost and potential stigma as long as CBR was helpful.

**Table 2:** Process data

Process data	Mean (range)
Months CBR received	11.2 (8,12)
Number home visits	21.1 (17,27)
Number optional goals achieved/selected^a^	5.5/8.9 (62%)
Number meetings with community members	2.4 (0,10)
Number referrals to health centre/ HEW	1.6 (0,4)

^a^All participants achieved all four compulsory goals

*“I didn’t worry about shortage of time, because [CBR] was very useful for me…It is for our own good. I would be very happy to learn the whole day, let alone for two hours”.*

(caregiver-1-E)

However, some participants disengaged for several weeks whilst they were visiting relatives or at holy water sites. One CBRW felt that the tapered reduction to monthly home visits made engagement harder to maintain as the intervention progressed. For three participants, clashes over the CBRWs’ recommendation to take medication contributed to unwillingness to participate in CBR. These participants felt that CBRWs ignored valid concerns about side effects and ‘nagged’ them to continue medication. These issues were sometimes resolved by minimising the emphasis on medication, but in one case there was a breakdown of trust that contributed to the participant stopping CBR after eight months.

*“It is boring when such advice is given repeatedly. I think the advice you got once could help you in your life…When [the CBRW] nags me repeatedly, I tell him that I don’t want to continue [CBR]*. *....He always created stress in my life.”*

(woman with schizophrenia-4-E)

A further two participants withdrew at 11 months, both of whom had improvements in functioning. In one case CBR was terminated because the participant moved away for work. A minority of participants complained that CBR interrupted their work, with the potential to lose customers or neglect time-dependent farming tasks.

*“When I am at the farm with the oxen…I feel irritated when [the CBRW] came because thirty minutes is too much for me…The farm work is a tiresome work as it needs much dedication…Unless the discussion with her is after the harvest work, it will be too long”.*

(man with schizophrenia-1-E)

It was often easier for female participants to take part as they could do domestic tasks, such as handicrafts, whilst talking with the CBRW. In several cases CBRWs successfully adjusted visit timings or frequency to fit in with participants’ schedules. Nevertheless, there were some complaints that CBR sessions were boring or easily forgettable. Several participants requested written materials to refer back to. Only one participant indicated that home visits could be stigmatising. Several CBRWs reported initial difficulties finding a caregiver willing to engage in CBR. Whilst for some this was resolved with careful negotiations, two participants had minimal family input.

Most CBR participants did not report any negative effects of community mobilisation. However, one man was unhappy with community awareness-raising, suggesting this could result in interference from outsiders. One optional activity, for people with schizophrenia to describe their experiences in a public forum, was not carried out in any sub-district. This was due to difficulty identifying a participant who was confident to speak in public.

#### Assumption 1b: CBR can address the needs of people with schizophrenia

All participants achieved all core goals. Participants selected a mean of nine optional goals (range 6–11), of which a mean of 5.5 (62%) were achieved (range 13–100%) (Table 2). There was a tendency to select more goals than would be achievable in 12 months. Initially the caregivers of the participants with co-morbid intellectual disabilities reported high hopes for change. These families expected complete recovery after a few weeks of medication, and were later disappointed.

Most participants demonstrated agency, disengaging if the service did not meet their needs, and articulating concerns in interviews. There was an almost unanimous expectation of financial support or free medication from CBR, despite explanations to the contrary at recruitment. This expectation reportedly arose from experience of non-governmental organisation hand-outs and a time compensation payment at baseline data collection. Given the severe poverty experienced by many participants and the out-of-pocket payment required for medication, the inability of CBR to provide financial support created significant disappointment and confusion. Several CBRWs spent the first few weeks negotiating to continue CBR and participants generally accepted what CBR could offer. However in one case these issues contributed to the participant withdrawing.

#### Assumption 1c: CBRWs are willing to work with people with schizophrenia

CBRWs were willing, and sometimes highly motivated, to work with people with schizophrenia. There were 220 initial applicants and no CBRWs left the project. Reported attitudes towards people with schizophrenia improved as the pilot proceeded, including lowered fears of violence and increased expectations of recovery.

***“*** *my perception of a mentally ill person has changed after I visited [the CBR participant]…My confidence was very little at the start. I used to think that mentally ill people always carried daggers to hurt people. But when I entered the house, I understood that he is not that kind of person.”*

(CBRW-8-E)

However, two CBRWs noted that whilst not all people with schizophrenia are violent, there are cases where this is true, and procedures to ensure CBR safety were felt to be important by supervisors. Some CBRWs and supervisors described stressful situations and also sadness at difficult circumstances. Peer support, during group supervision and also informally, was appreciated as a chance to gain new perspectives on how to overcome problems, or simply to derive relief from discussing issues.

#### Assumption 1d: Community leaders are willing to support CBR without benefits for themselves

Community leader meetings were difficult to arrange but were held in all sub-districts. Some community leaders were too busy or difficult to contact, whilst others were suspicious that the project was related to a political party. Several CBRWs felt community leaders were expecting financial benefits from participation, which was resolved by piggy-backing the meetings onto existing sub-district gatherings. Awareness-raising events were held in all sub-districts, at the sub-district council, Women’s groups, *edir* (traditional support association) groups and development meetings, with up to 100 participants (Table 2). CBRWs were generally well received within the community.

Targeted mobilisation of community figures was undertaken for six participants, including with a priest, government officials, an agricultural worker, health extension worker, *edir* leader and businessmen. All three community members interviewed had sacrificed time and money, but none perceived this as a hardship. Instead, giving support was portrayed as a gratifying experience, especially when improvements were seen.

*“You feel happy when you help someone whose economy is below you. When you see improvement in the person you are helping, you will be satisfied. I am very glad since I have helped him. I got happiness.”*

(male benefactor-7-E)

#### Assumption 1e: Traditional healers are willing to engage in CBR

Whilst priests and other religious leaders were identified, no traditional or religious healers were identified during resource mapping by CBRWs. Two CBR participants visited holy water sites, but these were located outside of Sodo district. There was therefore little potential for CBR engagement.

### Is the RISE CBR intervention feasible?

#### Assumption 2a: Non specialists can be trained to deliver CBR for schizophrenia

Competence varied between CBRWs but overall was good. CBRWs completed the required assessments for all participants and undertook 91.7% of indicated modules, suggesting ability to select appropriate CBR components. Many participants considered CBRWs as experts, who were able to explain clearly and give constructive advice. Most CBR participants described the pleasant manner of the CBRWs, as well as appreciating their listening skills (*“she asked me how I am feeling and I told her what I really feel*” (caregiver-1-B)). Whilst some weaker areas were identified (problem-solving, risk assessment and response, and giving advice without lecturing), in general problems with competence were not a barrier to CBR delivery.

#### Assumption 2b: CBRWs can overcome logistical issues

Practical challenges included the long distances on foot between households and patchy public transport. Whilst these were largely manageable in the pilot, there were concerns this would be problematic when CBR was delivered on a larger scale. Problems with the phone network made it difficult to arrange home visits and supervision meetings. Despite this there was strong support for home visits from CBRWs and supervisors to encourage engagement, understand the family environment and give advice to the extended family.

#### Assumption 2c: Primary care staff are supportive of CBR

CBRWs were advised to accompany participants to primary care appointments around the time they conducted each of the three periodic CBR needs assessments. In the pilot, CBRWs accompanied participants to primary care a mean of twice over 12 months (range 1–3 times). Some participants appreciated support in discussing their treatment with health officers. CBRWs made fourteen referrals to the health centre, relating to eight participants, for medication reviews due to side effects, physical illness, relapse and suicide risk. Health centre staff described a productive working relationship with CBRWs, which enhanced the quality of facility-based care.

*“I think [the CBRWs’] presence is useful for us to give better treatments to patients…We have a very good relationship…they directly come to us when they encounter problems and need our help.”*

(health officer A)

#### Assumption 2d: Anti-psychotic medication is accessible

The majority of CBR participants reported that medication, particularly depot injection, was unaffordable.

*“Now the main problem is the boy refuses to take the [oral] medicine and the injection is expensive and I am poor and a newcomer to the town, I have no one to help me...the medicine had [brought] improvement. Now my problem is the money to buy the medicine…The boy who was having improvement is going to be ill.”*

(caregiver-8-B)

None of the four CBRWs who endeavoured to obtain the medication fee waiver were successful. Several participants reported frustration at medication being unavailable at the health centre or not being dispensed due to a lack of pharmacist, receipts, or because the caregiver was not present. All participants reported medication side-effects, most commonly drowsiness and weakness. These effects were frequently intolerable, particularly due to the impact on physical labour required for farm work. Side-effects resulted in interrupted adherence (particularly during harvest time) despite medication also conferring benefits, according to some participants. Some stopped once they felt well, or because they saw no positive impact. One CBR participant interrupted medication whilst at holy water, believing the two treatments were not compatible and another stopped medication altogether partly due to her family’s belief that the illness was caused by spirit possession.

#### Assumption 2e: *Edir* support will be available and sustainable

There were no examples of practical or financial support for CBR participants from *edir* (traditional support association) groups. CBR workers highlighted that this was an optional component of CBR that had not been emphasised in the training. Where involvement was attempted, there was a lack of firm interest or engagement from *edir* groups or in other cases people with schizophrenia or families did not wish to receive support from such organisations. Edir groups were however used as a vehicle for awareness-raising.

### Can the RISE CBR intervention produce an impact and if so, how?

#### Assumption 3a: CBR can improve functioning in people with schizophrenia

##### Changes in functioning

Most CBR participants began the study with high levels of disability (baseline median WHODAS 57.5 (IQR (interquartile range) 36.7, 65.1), which decreased over 12 months (endline median WHODAS 18.4 (IQR 2.4, 46.2)) (Table 3). The two women with co-morbid intellectual disability saw little improvement. Amongst those with improvements, changes were seen in work, domestic activities, social participation and self-care, though several barriers to achieving change were identified. In general there was consistency between qualitative and quantitative data.

**Table 3:** Individual disability (WHODAS) scores

CBR participant ID	Disability (WHODAS total)		
	0 months	6 months	12 months
1	21.7	0	67.0
2^a^	75.5	80.2	76.4
3^a^	64.2	95.3	Participant died
4	54.7	22.6	4.7
5	65.1	56.6	25.5
6	36.8	17.0	Participant declined
7	32.1	34.0	0
8	69.8	12.3	18.9
9	49.1	5.7	0
10	60.4	43.4	17.9
Median (interquartile range)	57.5 (36.7, 65.1)	28.3 (12.3, 56.6)	18.4 (2.4, 46.2)

^a^Co-morbid intellectual disability

Some CBRWs bemoaned the lack of formal employment opportunities for their clients. Yet CBRWs were able to support five participants to start new income-generating activities, including daily labouring, selling farm produce, handicrafts and local alcohol production, whilst two participants resumed existing work. All types of participants felt participating in livelihood activities to be the most important possible change. Greater participation in household tasks was often reported and two female participants began actively caring for their children. Some participants began leaving the house unaccompanied, whilst others began participating in important social activities such as coffee ceremonies or attending church, weddings and funerals. Self-care reportedly improved in all eight participants where this had been a problem. Although several CBR participants felt sad when CBR finished, most felt confident they could maintain their progress. In contrast, several CBRWs were pessimistic that positive impacts would be sustained due to the long duration of illness and difficulty maintaining motivation from the caregivers.

##### How CBR improves functioning

**Fig. 3:**
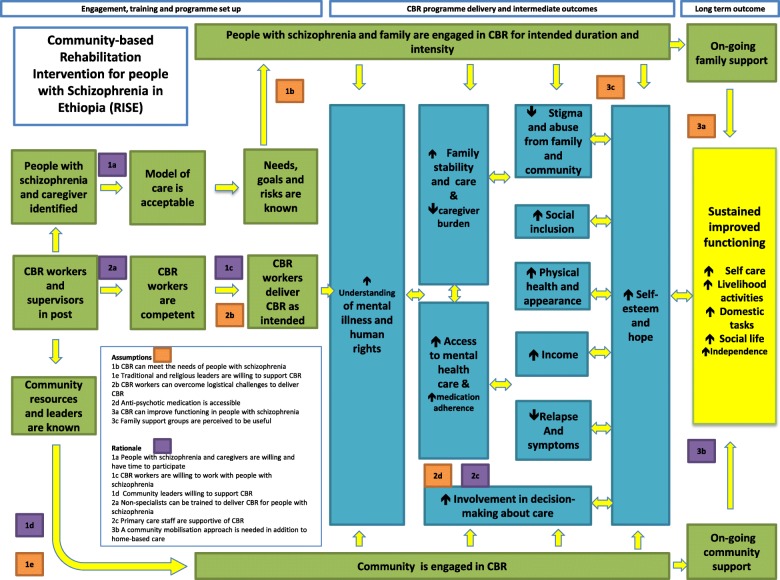
Post-pilot theory of change

The perceived pathways to improved functioning differed between participants. Both antipsychotic medication and CBR at the individual, family and community level had varying degrees of influence. However, general conclusions can be drawn (Fig. 3 blue boxes).

#### Increased understanding of mental illness and human rights

Several participants reported an improved understanding of the symptoms, causes, and basic rights of people schizophrenia, and appreciation of the potential benefits of medication.

#### Increased family stability and care

Involving family members in CBR was seen as central to improving functioning. Several CBRWs noted that until disruptive and strained family environments were stabilised, improvements were difficult. In several instances CBRWs acted as mediators in family conflicts, leading to individuals being able to return home and therefore addressing basic needs of shelter and food.

*“[the man with schizophrenia] was expelled from home because he kicked his mother….He was roaming the streets and was unable to stay at home because of his illness…But the family relationship improved significantly after I gave them the lesson from the module about interpersonal relationships in the family...he asked for her forgiveness and they started living together happily.”*

(CBRW-8-E)

Most caregivers described strategies they had learnt to avoid aggravating their relatives, including communicating calmly, avoiding stressors and telling them of plans in advance. Some caregivers began facilitating self-care, for example by buying their relative soap. Caregiver burden scores reduced between baseline and six months, and then plateaued at 12 months (Table 4 and Additional file 8). Several caregivers described relief from constant worry. There was less need to supervise their relative meaning that daily tasks could be completed. Others welcomed the increased contribution to communal work.

**Table 4:** CBR participant outcomes

	Median (IQR)		
Outcome	Month 0 (n=10^a^)	Month 6 (n = 10)	Month 12 (*n* = 8)
Clinical global impression (% normal/borderline)	0%	50%	62.5%
Discrimination (DISC-12 total)	2 (0,4)	0 (0,4)	0 (0,3.5)
Depression (PHQ9 total)	10.5 (6,13)	6 (2,11)	3.5 (1.5,8.5)
Alcohol use (AUDIT total)	3.5 (0,7)	4.5 (2,13)	2.5 (0,5.5)
Caregiver burden (IEQ)	46 (37,61)	26.5 (21,48)	30.5 (19,41.5)

^a^n = 9 for Clinical Global Impression and IEQ

#### Increased access to mental healthcare and anti-psychotic medication adherence

CBRWs showed participants where and how to buy medication, reminded them to attend follow-up and escorted them to appointments. Whilst initially there was optimism that this support alone would be sufficient, unaffordability of medication later emerged as an important barrier. In three cases, CBRWs addressed this by facilitating local benefactors or family members to purchase medication. Family members also learnt how to recognise and respond to relapse and to remind their relative to take medication.

#### Decreased relapse and symptoms

The proportion assessed as normal or borderline on the Clinical Global Impression rose from zero at baseline to 62.5% at endline (Table 4 and Additional file 8). Many participants felt that simply taking medication had made individuals ‘well’. Whilst a priest spoke of one man with schizophrenia gaining a ‘peaceful and free mind’ through treatment, for most CBR workers and caregivers being healthy was associated with a reduction in behaviours such as collecting objects, throwing stones, wandering away from home and talking to themselves. In many cases, improvements in symptoms led to better functioning. Median depression scores decreased over the pilot (Table 4 and Additional file 8). A ‘see-sawing’ effect was seen in two participants, with scores either much higher or lower at six months, compared to baseline and endline. It was difficult to directly tally these patterns with the participants’ accounts in qualitative interviews. Several people with schizophrenia reported feeling calmer or better able to cope with suicidal thoughts. This was attributed to improvements in the family environment or their functioning, or taking medication, but also to new coping strategies.

***“*** *I used to get depressed when I sit at home. I used to cry and go outside the house. These things have reduced now. I have built my own house and have started making my bed and living like other people….When I was sitting alone at night, I used to think that it would be better if I hanged myself or threw myself into a river than live like this...But I have significant improvements after [the CBRW] advised me to go outside, watch TV and relax, when I feel low and bad ideas come to my mind.”*

(man with schizophrenia-5-E)

#### Increased income

Participation in livelihood activities brought various benefits: improved self-esteem, reduced caregiver burden, a shift in community attitudes, but also income. Increased income in turn brought the ability to pay for medication, to support the wider family and to make financial contributions to community organisations.

*“What I did for my patient was to make her do the job she was doing in the past. So, she started distilling araki [local gin] to cover her household expenses. Doing some work will help them generate income and they will be happy because they will be able to do whatever they want. They will not expect anything from anyone…the community will start thinking that they can take care of themselves and do some work.”*

(CBRW-10-E)

#### Improved physical health and appearance

CBRWs had some successes in supporting physical wellbeing. Two people with schizophrenia were referred to the health centre for musculoskeletal problems that were preventing farm labouring. However one woman with schizophrenia, whose family were resistant to the CBRW’s efforts to facilitate treatment, ultimately died of an undiagnosed physical illness. Quantitative data indicated that three of the four participants who exhibited problem drinking cut down (Table 4 and Additional file 8). Whilst there were indications from the qualitative data that CBRW advice played a role in some cases, in one case the CBRW felt problem drinking had not reduced over time.

#### Increased social inclusion

There were reductions in reported discrimination for most participants (Table 4). At baseline, seven of the participants reported any experience of discrimination; by endline only the two participants who initially had the highest scores continued to report discrimination (Additional file 8). For some, non-participation in social activities was a personal choice but others reported social exclusion due to the stigmatising attitudes of community members. The priest described how a change in attitudes had reduced social exclusion.

*“Before, [the man with schizophrenia] was considered to be crazy and he wasn’t allowed to participate in any activities in the community. But today he participates in activities in the community. They take care of him…He is healed and today he is healthy and is now working…he wasn’t invited to weddings, or attend funerals...However, today the community supports him, the community has embraced him.”*

(priest-5-E)

Notably the account of the man supported by this priest differed from the priest’s own account. For this man, re-integration was problematic due to persistent negative perceptions from community members. According to this man, CBR had not assisted with the social exclusion he experienced; this was also reflected in his

DISC score, which remained high at the end of CBR, despite some improvement.

*“The medication and the [CBR] education have helped me a lot in my recovery [...] But, I have to start a social life […] I don’t go to anyone’s funeral and no one will bury me if I die […] No one invites me because I am living alone and I don’t have social life […] I am lonely.”*

(man with schizophrenia-5-E)

#### Decreased stigma and abuse

Some caregivers began treating their family members with dignity or ensuring they had sufficient food, and in one case physical abuse ended. There were two instances of physical restraint (of one and three days duration). In one case the CBRW resolved the situation by negotiating with the caregiver and encouraging access to treatment. One participant described learning strategies to deal with stigmatizing comments.

#### Increased self esteem and hope

Increased self-esteem and hope seemed to underpin sustained changes in functioning; whilst functioning – whether participation in work, social life or improved self care - fostered a feeling of self-worth. For some the experience of receiving support at all, and therefore feeling valued, appeared to have an independent influence on wellbeing. Often the knowledge that the illness could improve was transformational, whilst some CBRWs were great motivators. Self-esteem manifested in various ways including participants taking pride in their appearance and work, feeling equal to others, and being assertive.

*“When he eats he wants to get good food…when he goes to parties he doesn’t accept leftovers, he wants good food and he wants to be seated and served like normal people. He says he is not less than anyone, he is fine.”*

(caregiver-7-E)

#### Assumption 3b: A community mobilisation approach is needed in addition to home-based care

Many felt that CBR had increased public understanding about the signs and treatability of mental illness. Several participants suggested that visibly improved functioning in people with schizophrenia was a turning point in changing attitudes. There was agreement that improved community attitudes can potentially impact on mistreatment and social exclusion. However, few participants, and no people with schizophrenia, provided concrete examples of awareness affecting inclusion. Several participants reported that awareness-raising meetings had led to local people with psychosis accessing treatment for the first time. There were four participants for whom CBRWs mobilized tangible community support. Support included provision of a house, food and medication; identification of employment opportunities; moral support; family mediation; advocacy for a medication fee waiver; a small business loan; and support with medication and social activities. Those providing support had minimal previous involvement with the CBR participant, which had been enhanced and formalized by CBR.

*“I used to feel very sad when I saw [the man with schizophrenia] in the street…I used to give him some food or some small things and encourage him. I was giving him some unsustainable support…There was no fixed thing. I used to forget him and pass him. But now there is someone in the middle who can ask for him and arrange for us to meet.”*

(benefactor-7-E)

CBRWs sometimes faced difficulties finding benefactors anywhere except in the wealthier urban areas, and sustaining support was problematic.

#### Assumption 3c: Family support groups are perceived to be useful

Only one sub-district set up a family support group, which ended after three meetings. The lack of a saving and loans element was not given as a reason for non- participation. Instead participants felt uncomfortable discussing personal issues or their relative was too ill to be left unattended.

### Revised theory of change framework

A revised post-pilot theory of change framework was created (Fig. 3). Assumptions that were met were converted to rationales (purple boxes). Assumptions that were not met were retained (orange boxes). A revised conceptual framework for how CBR impacts on functioning was included (blue boxes).

## Discussion

### Strengths and limitations

This pilot study produced rich insights on the acceptability, feasibility and potential impact of CBR for schizophrenia in Ethiopia over 12 months. A strength was the use of several sources of qualitative data to enhance credibility of the findings. Triangulation laid bare discrepancies between narratives, with people with schizophrenia tending to offer less positive accounts of CBR. This highlights the importance of incorporating service user perspectives, including those who have disengaged, in the evaluation of mental health interventions. The trustworthiness of the findings was also established through researcher debriefing, vetting of sub-themes by team members, and testing themes against raw data [38]. Another strength was the systematic use of theory of change; we identified and tested assumptions for how the intervention will work, and on the basis of these findings refined the intervention for evaluation in a cluster-randomised trial [19]. Both the pilot intervention and evaluation design were responsive, allowing emerging questions to be investigated, and the findings to be acted upon. The inclusion of participants with co-morbid intellectual disability was a pragmatic design decision that reflects the likely range of conditions that would be included at the implementation stage of CBR for schizophrenia. This therefore improves the generalisability of the results. We anticipate the RISE materials [30] will be relevant for researchers, NGOs and policy-makers in Ethiopia and other LMICs aiming to deliver community-based support for people with schizophrenia whilst using limited resources.

There are some limitations to this study. First, there is a likelihood of social desirability bias as the investigators and some respondents had a vested interest in the intervention’s success. This may have been compounded by low expectations amongst CBR participants; the “sense that ‘any mental health care’ was something to be grateful for” has been identified in rural Ethiopia [33]. These concerns are somewhat allayed by the criticisms of CBR that some people with schizophrenia voiced. Furthermore, qualitative data showed good consistency with quantitative. Second, this study does not allow us to make conclusive judgements on the impact of CBR. The role of the RISE trial will be to determine the effectiveness of the finalised CBR intervention on functioning and to elucidate the potential mediating role of medication adherence [19]. Third, CBRWs only had one client each, compared to an anticipated eight clients in the RISE trial. The low workload may have concealed feasibility issues and also led to more intensive, and potentially more effective, intervention delivery.

### Intervention modifications

The initial engagement phase was extended from two to three months, to give time for building relationships and clarifying what CBR could offer. A better understanding of how to access the medication fee waiver was established. *Edir* support was removed as a CBR strategy; instead efforts were focused on mobilizing support from the wider community. CBRWs were encouraged to alter the duration, timing and location of visits to fit with participants’ needs, and telephone contact between monthly visits became standard. CBRW refresher training covered problem solving and the balance between encouraging medication adherence and accepting the participant’s wishes. Intervention forms, such as needs assessments, were amended iteratively to improve usability. Written materials were created for participants. CBRW workload in the trial was reduced from twelve to eight participants.

### Conceptual framework for improved functioning

This study found that overall the RISE CBR intervention may have a positive impact on functioning. Qualitative and quantitative data supported this finding. A heightened understanding of mental illness and human rights was reported to have led to enhanced family support and increased access to health care. These changes paved the way for decreased stigma, increased social inclusion, improved physical health, reduction in symptoms, and increased income. Improved functioning appeared to be sustained by improved self-esteem and vice versa.

The pilot findings prompted four major changes to the conceptual framework (compare Figs. 2 and 3). First, bi-directional arrows were introduced throughout in recognition that there is rarely a set sequence of intermediate outcomes that produce improved functioning and that most outcomes positively affect each other in a recursive manner. Second, increased hope and self-esteem was a new intermediate outcome. That CBRWs recognised the importance of, and managed to increase, hope amongst participants indicates a recovery-oriented approach. However, CBRWs sometimes struggled to apply other recovery principles, such as collaborative goal-setting. Third, the intermediate outcome ‘The family can cope’ became ‘Increased family stability and care’. This reflects the powerful influence that family environment has on the person with schizophrenia, rather than perceiving families as simply passive victims of the illness. There were indications that CBRWs successfully supported families to reduce expressed emotion and that this brought palpable benefits. Families were central to the success of CBR, implying that the person with mental illness and their family should be considered the unit of intervention delivery in the Ethiopian context [13]. Fourth, ‘Increased involvement in decision-making about care’ became an intermediate outcome. Excessive persuasion to take medication represented one of the least acceptable elements of CBR. Respecting the wishes of people with schizophrenia, despite the limited treatment choices, was discovered to be important to encourage engagement and minimize stress.

### Assumptions found to be correct

Six assumptions were judged to be well founded and converted to rationales in the theory of change (Fig. 3 purple boxes). Three of these rationales related to acceptability (*rationale 1a, 1c and 1d*). As in similar studies, concerns about the safety of CBRWs delivering home care to people with schizophrenia were raised during planning, but were less of an issue in practice given appropriate supervision [16]. Challenges relating to acceptability from the perspective of people with schizophrenia were felt to be surmountable with supplementary CBRW training and increased flexibility in CBR delivery. Potential stigma was a concern for a minority of participants, similar to findings from India [16]. Disengagement due to time constraints was more significant. The confirmation that non-specialists can be trained to deliver CBR for schizophrenia (*rationale 2a*) supports findings from India [11]. That in the RISE pilot study this was achieved without routine psychiatrist supervision could represent a significant innovation, partly attributable to the structured nature of the intervention.

### Assumptions not yet confirmed

Seven assumptions could not be confirmed. First was the assumption (*1b*) that CBR can meet participants’ needs. Poverty was the foremost problem for participants; the lack of financial benefit from CBR was a key acceptability issue, prompting several participants to question the purpose of participation. Whilst expectations may be low, this did not translate into unquestioning engagement if needs were not met. The absence of social security or local non-governmental organisations meant participants were wholly reliant on the CBR programme to meet their needs. Support from local benefactors was seen as precarious, requiring intensive input and only available in wealthier urban areas. In many rural areas there may be rich community resources in terms of social and cultural life [24], but few ‘spare’ material resources. There was also a lack of interest from existing social organisations, such as *edir* (burial association) groups, in providing financial support (*assumption 2e*). Previous research has found that due to a lack of financial capital, financial support or lending to vulnerable or sick individuals is offered by a minority of edir groups [39], or not at all [40]. Stigma towards people with mental illness may further reduce the likelihood of support being offered. Some participants expressed disinterest in receiving support from *edir*; perhaps a more discreet means of support would have been more attractive. There was little firm data to fully explore this issue. Further emphasis and practical training on *edir* engagement may also have been beneficial for CBR workers. For whatever reason this arose, the inability to link CBR to a permanent social forum, as recommended by the WHO CBR guidelines [10], may have important implications for sustainability. Attempts by CBRWs to access the medication fee waiver were also unsuccessful. Though there were successes in supporting income-generation, important challenges also emerged including a lack of formal employment opportunities.

A key threat to the feasibility of CBR was the absence of continuously available and affordable medication with an acceptable side-effect profile (*assumption 2d*). Difficulties paying for medication and intolerable side-effects and have been identified previously as barriers to sustained engagement with mental healthcare in rural Ethiopia [41, 42]. To maximise the potential impact of CBR, robust systems are needed to ensure there is continuous provision of psychotropic medication to primary care. Anti-psychotic medication should either be made free or available through a health insurance scheme. The logistics of intervention delivery (*assumption 2b*) require further evaluation at scale. There are indications that CBR can improve functioning (*assumption 3a*), but the RISE trial results will determine effectiveness. A larger pool of participants will allow us to evaluate the utility of family support groups (*assumption 3c*) and acceptability of CBR to traditional healers (*assumption 1e*). The lack of engagement with traditional healers was surprising given evidence there are many operating in Sodo district [24]. Traditional healers may not have been identified by CBRWs because this group is hidden from public life or due to reluctance by CBRWs.

## Conclusion

In LMIC such as Ethiopia, CBR may be an acceptable and feasible adjuvant service to facility-based care for people with schizophrenia. CBR may have the capacity to improve functioning through supporting livelihoods, maximising family and community support, facilitating access to anti-psychotic medication, and increasing hope. However, contextual factors, including poverty and inaccessible anti-psychotic medication, remain substantial challenges. Some CBRWs found it challenging to accept the choices of people with schizophrenia. Greater emphasis on the involvement of people with schizophrenia in decision-making may tackle this issue.

## Additional files

Additional file 1: Trial procedures. Word document. Method and results for piloting of trial procedures. (DOCX 12 kb)

Additional file 2: CBR worker competencies. Word document. List of CBR worker competencies and assessment. (DOCX 15 kb)

Additional file 3: Overview of RISE CBR intervention. Word document. Summary of structure and content of RISE intervention. (DOCX 117 kb)

Additional file 4: Topic guides. Word document. Topic guides for 2 month and endline qualitative work. (DOCX 29 kb)

Additional file 5: Instruments. Word document. Further details on quantitative data collection instruments. (DOCX 21 kb)

Additional file 6: Assumptions table. Word document. Summary of main findings, adjustments to intervention and conclusion relating to each theory of change assumption. (DOCX 20 kb)

Additional file 7: Supplementary qualitative data. Word document. Additional supporting quotations from qualitative data, by theme and sub-theme. (DOCX 42 kb)

Additional file 8: Individiual level process and outcome data. Word document. (DOCX 17 kb)

## Abbreviations

AUDIT: Alcohol Use Disorders Identification Test
CBR: Community-based rehabilitation
CBRW: Community-based rehabilitation worker
DISC-12: Discrimination and Stigma Scale-12
FGD: Focus group discussion
IDI: In depth interviews
IEQ: Involvement Evaluation Questionnaire
LMIC: Low and middle income countries
LSHTM: London School of Hygiene and Tropical Medicine
PHQ-9: Patient Health Questionnaire-9
PRIME: Programme for Improving Mental healthcarE
RCT: Randomised controlled trial
RISE: Rehabilitation Intervention for people with Schizophrenia in Ethiopia
ToC: Theory of Change
WHODAS: World Health Organisation Disability Assessment Schedule
