# Supplementary material for: Community-based rehabilitation intervention for people with schizophrenia in Ethiopia (RISE): a 12 month mixed methods pilot study
Source: BMC Psychiatry. 2018 Aug 3;18:250. doi: 10.1186/s12888-018-1818-4 (PMC6091097; doi:10.1186/s12888-018-1818-4)
Supplement: Supplementary file 1 — Trial procedures. Word document. Method and results for piloting of trial procedures. (DOCX 12 kb) [file 12888_2018_1818_MOESM1_ESM.docx]

**RISE pilot: trial procedures**

**Methods**

Eligibility and consent procedures, adverse event detection, process and quantitative data collection and management were piloted. Attrition rates were also estimated. Data from previous local studies were used for the trial power calculations.

**Results**

Due to instances of disengagement from CBR and refusal to participate in endline data collection the estimated attrition rate was increased from 15% to 23%. Out of 17 participants screened, 10 were eligible. Due to lower than expected numbers of PRIME cohort recruits with RISE-eligible diagnoses a lower threshold was given for the criterion ‘Evidence of enduring or disabling illness’ in the trial (CGI threshold 3 instead of 4). Out of 10 eligible participants, all 10 consented to participate in the pilot. No changes were made to consent or adverse event procedures. Minor modifications were to process data collection forms to increase ease of use. Additional training was given to data collectors and data entry clerks on the basis of minor problems observed by research staff.
