# Supplementary material for: Community-based rehabilitation intervention for people with schizophrenia in Ethiopia (RISE): a 12 month mixed methods pilot study
Source: BMC Psychiatry. 2018 Aug 3;18:250. doi: 10.1186/s12888-018-1818-4 (PMC6091097; doi:10.1186/s12888-018-1818-4)
Supplement: Supplementary file 2 — CBR worker competencies. Word document. List of CBR worker competencies and assessment. (DOCX 15 kb) [file 12888_2018_1818_MOESM2_ESM.docx]

**CBR worker competencies**

| **1** | **Competency** | **Assessment** |
| --- | --- | --- |
|  | **Administrative** |  |
| 1a | Able to complete documentation correctly | Role play |
| **2** | **Professionalism** |  |
| 2a | Aware of and adheres to cultural norms in terms of dress and general behaviour | Observation by trainers |
| 2b | Demonstrates good time-keeping and attendance | Observation by trainers |
| 2c | Demonstrates positive attitude (i.e. not stigmatizing) towards people with schizophrenia | Observation by trainers |
| 2d | Has skills to maintain own wellbeing | Observation by trainers  Written test 1 |
| **3** | **Communication skills** |  |
| 3a | Able to build trusting relationship with person with schizophrenia and their family | Role play |
| 3b | Able to deal with difficult situations e.g. angry person, violent person | Written test 1 |
| 3c | Has good listening skills | Role play |
| 3d | Is able to explain concepts clearly, check for understanding etc | Role play |
| 3e | Can employ a problem solving approach | Role play |
| **4** | **Referrals** |  |
| 4a | Aware of circumstances in which to contact supervisor for support | Role play  Written test |
| 4b | Aware and able to follow procedures for the following scenarios: suicidal intent, neglect, etc | Role play  Written test |
| 4c | Aware and able to follow procedures for following scenarios: identification of physical or mental illness in community member | Role play  Written test |
| 4d | Aware of health services available for people with mental illness | Role play  Written test |
| **5** | **Assessment and review** |  |
| 5a | Able to assess achievement of goals on an ongoing basis | Role play  Written test |
| 5b | Able to conduct a needs assessment for people with schizophrenia and caregivers in conjunction with supervisor | Role play  Written test |
| 5c | Able to detect distress in caregivers on an ongoing basis | Written test 1 |
| 5d | Able to conduct a brief risk assessment | Role play |
| 5e | Able to conduct goal setting and develop a rehabilitation plan in conjunction with supervisor | Role play |
| **6** | **Knowledge** |  |
| 6a | Has basic knowledge of mental disorders including depression, alcohol use disorder etc | Written test |
| 6b | Has good knowledge of schizophrenia (causes, course) and medical treatment; and can identify people with schizophrenia | Written test |
| 6c | Understands the impact of schizophrenia in terms of disability, stigma, human rights abuses and family burden | Written test |
| **7** | **CBR delivery** |  |
| 7a | Understands and is able to explain purpose and structure of CBR programme | Role play |
| 7b | Understands structure and purpose of RISE pilot and RISE trial | Written test 1 |
| 7c | Able to give information on schizophrenia | Role play |
| 7d | Able to assess reasons for not accessing medication or attending health centre and apply problem solving approach to address this | Role play |
| 7e | Able to give information on how to deal with a crisis and support development of crisis management plan | Written test |
| 7f | Able to assess reasons for not taking medication and apply problem solving approach to address this | Role play |
| 7g | Able to identify early warning signs with family and develop relapse prevention plan | Written test 2 |
| 7h | Able to develop plan to enable improved self-care and improve participation in household tasks | Written test 1 |
| 7i | Able to deliver family intervention | Written test 1 |
| 7j | Able to sensitively address chaining and physical abuse and take appropriate steps to address this | Written test 2 |
| 7k | Able to give advice for dealing with distressing symptoms | Written test 1 |
| 7l | Able to give advice for dealing with stress and anger | Written test 1 |
| 7m | Able to give advice to improve healthy behaviours | Written test 1 |
| 7n | Able to assess reasons for poor physical health and use problems solving to address this | Written test 2 |
| 7o | Able to give advice on dealing with stigma and discrimination | Written test 1 |
| 7p | Able to assess reasons for reduced participation in community life and apply problem solving approach to address this | Written test 1 |
| 7q | Able to assess reasons for reduced participation in vocational activities and apply problem solving approach to address this | Written test 2 |
| 7r | Able to assess literacy and basic skills suggest steps to improve them | Written test 1 |
| 7s | Able to transfer skills to caregivers in order for family to continue CBR after end of programme | Written test |
| 7t | Aware of procedures for ending intervention with family | Written test 1 |
| **8** | **Community mobilisation** |  |
| 8a | Able to form good relationships with community leaders and HEWs | Written test 1,2 |
| 8b | Able to conduct community awareness-raising | Written test 2 |
| 8c | Able to engage with community leaders to facilitate CBR | Written test 1,2 |
| 8d | Able to maximise sustainability of programme through community involvement | Written test |
| **9** | **Family support group** |  |
| 9a | Able to organise and facilitate support groups or befriending arrangements | Written test 1,2 |
