# Supplementary material for: Community-based rehabilitation intervention for people with schizophrenia in Ethiopia (RISE): a 12 month mixed methods pilot study
Source: BMC Psychiatry. 2018 Aug 3;18:250. doi: 10.1186/s12888-018-1818-4 (PMC6091097; doi:10.1186/s12888-018-1818-4)
Supplement: Supplementary file 3 — Overview of RISE CBR intervention. Word document. Summary of structure and content of RISE intervention. (DOCX 117 kb) [file 12888_2018_1818_MOESM3_ESM.docx]

**Overview of RISE CBR intervention**

| **Phase** | **CBR Review** | **Goals** | **Modules** | **Community engagement tasks** | **Family Support Group** |
| --- | --- | --- | --- | --- | --- |
| **I** | CBR Review I:   - Initial Assessment - Needs Assessment - Goal setting for Phase I - Risk Assessment - Accompany to health centre - Rehabilitation plan | Individual and caregiver have been informed of what schizophrenia is, available treatments, and the potential for recovery | Understanding schizophrenia and its treatment | Task 1: Meet with health extension worker/s  Task 2: Identify key community leaders  Task 3: Identify key community resources  Task 4: Ascertain what community engagement relating to mental illness has already taken place or is planned  Task 5: Meet with key community leaders  Task 6: Community awareness-raising  Task 7: Identify potential employment opportunities in the kebele |  |
|  |  | Individual is able to access medication  and attend health centre for mental health as indicated by clinical status | Improving access to health services |  |  |
|  |  | Crisis management plan is in place | Preparing for a crisis |  |  |
|  |  | Person with schizophrenia is not chained or restrained | Dealing with human rights issues |  |  |
| **II** | CBR Review II:   - Needs Assessment - Goal setting for Phase II - Risk Assessment - Accompany to health centre - Invite to Family Support Group - Rehabilitation plan | Individual is willing to take medication | Supporting individuals to take medication | Task 8: Individual meetings with Kebele leaders  Task 9: Individual meetings with Edir leaders  Task 10: Individual meetings with religious leaders  Task 11: Individual meetings with traditional healer/ holy water priest/ attendant  Task 12: Demonstrate progress of client/s to community leaders/ wider community  Task 13: Community awareness raising consolidation  Task 14: Facilitate employment opportunities in the kebele  Task 15: Individual meetings with literacy group leader | Support group active |
|  |  | Individual has strategies to remember to take medication |  |  |  |
|  |  | Individual feels side effects are improving |  |  |  |
|  |  | Individual feels hallucinations and delusions are improving | Dealing with distressing symptoms |  |  |
|  |  | Individual feels problems with motivation and thinking clearly are improving |  |  |  |
|  |  | Individual is able to access health services for physical and sexual health needs and contraception when required | Improving access to health services |  |  |
|  |  | Individual has strategies to deal with stress and anger | Managing stress and anger |  |  |
|  |  | Individual has information to make decisions about health-related behaviours | Improving Physical Health |  |  |
|  |  | Individual has good physical, sexual and reproductive health |  |  |  |
|  |  | Individual is not malnourished |  |  |  |
|  |  | Individual participates in community life | Taking part in community life |  |  |
|  |  | Individual participates in religious activities if they are important to the individual |  |  |  |
|  |  | Person with schizophrenia is able to interact socially with neighbours and friends |  |  |  |
|  |  | Individual has improving ability to do parenting activities | Improving the family environment |  |  |
|  |  | Individual can carry out usual family role |  |  |  |
|  |  | Individual has improved relationship with family members |  |  |  |
|  |  | Caregiver has improved ability to cope |  |  |  |
|  |  | Individual has improving self-care | Improving day to day functioning |  |  |
|  |  | Individual has improving ability to do household tasks |  |  |  |
|  |  | Individual has improving self-esteem | Dealing with stigma and discrimination |  |  |
|  |  | Individual does not feel discriminated against |  |  |  |
|  |  | Individual is not the victim of physical, sexual or emotional abuse | Dealing with human rights issues |  |  |
|  |  | Individual has restored participation in livelihood activities, including farm work | Getting back to work |  |  |
|  |  | Individual has basic literacy skills | Improving literacy |  |  |
| **3** | CBR Review III:   - Needs Assessment - Goal setting for Phase III - Risk Assessment - Accompany to health centre - Rehabilitation Plan | Individual has relapse prevention plan | Taking control of your health | Any Phase 2 Community Engagement task | Support group active |
|  |  | Any Phase 2 goal | Any Phase 2 module |  |  |
