# Supplementary material for: Community-based rehabilitation intervention for people with schizophrenia in Ethiopia (RISE): a 12 month mixed methods pilot study
Source: BMC Psychiatry. 2018 Aug 3;18:250. doi: 10.1186/s12888-018-1818-4 (PMC6091097; doi:10.1186/s12888-018-1818-4)
Supplement: Supplementary file 4 — Topic guides. Word document. Topic guides for 2 month and endline qualitative work. (DOCX 29 kb) [file 12888_2018_1818_MOESM4_ESM.docx]

**Topic guides for RISE pilot qualitative work**

**RISE pilot initial evaluation (2 months): Topic guide for IDIs with individuals with schizophrenia/caregivers**

1. **Tell me about your experience of being involved in CBR so far.**

- What were the best things
- What were the worst things

1. **How did you find the experience of being involved in CBR?**

Prompts

- What was it like being visited at home?
- How did you find the frequency of the visits?
- How did you find it when you were asked whether you had thoughts of harming themselves?
- How did you find it when you asked whether you had been chained up recently?
- What was it like being accompanied by the CBR worker to the health centre?

1. **How did you find the CBR worker?**

Prompts

- What is your relationship with the CBR worker like?
- How did you find their manner?
- What do you think about their knowledge?
- How were they at listening to you?
- How were they at explaining things?

1. **Is CBR useful for you so far?**

Prompts

- What problems and needs do you have? Does CBR address your needs? How?
- What expectations did you have of CBR when you started? Has it met your expectations? In what ways?
- How has CBR impacted on your understanding of schizophrenia?
- How has CBR impacted on your ability to access medication and the health centre?
- How has CBR impacted on how often you are chained up?
- How easy to understand was the information you received?
- How did you find the format of the information? (e.g. which is better: handouts or just hearing the information verbally?)

1. **What can we change or add to CBR to make it better for you?**

Prompts

- Have you had any problems taking part in CBR?
- If so, is there anything that can be done to make it easier for you to take part?
- Is there anything that would improve CBR?

**RISE pilot initial evaluation (2 months): Topic guide for FGDs with CBR workers/ IDI with supervisor**

1. **What has been your experience of being a CBR worker/supervisor so far**
2. **How useful do you think CBR is for the participants?**

**Prompts**

- What expectations do you think the individuals and families had of CBR?
- Do you think you will be able to meet those expectations?
- In what ways do you think your home visits are useful? Give me some examples from your experience.
- How has CBR impacted upon the individual’s understanding about schizophrenia?
- How has CBR impacted upon the individuals’ ability to access care?
- How has CBR impacted upon human rights issues such as chaining?

1. **What challenges did you have in your work?**

**Prompts**

- Was there anything that made it difficult for individuals and families to participate?
- Was there anything that made it difficult for you to deliver CBR?
- How did you overcome these challenges?
- Is there anything that can be done to make it easier for you to deliver CBR?
- Is there anything that can be done to make it easier for individuals and families to participate?

**FOR CBR WORKERs:**

1. **What was it like doing CBR home visits?**

**Prompts**

- What was it like working with a person with schizophrenia +/- intellectual disability?
- What is your relationship with the individual and family like?
- How confident do you feel in delivering CBR?
- How did you find asking about suicide risk?
- How did you find asking about chaining?
- How acceptable do you think it is to ask about these things every time?
- If you had to deal with any difficult situations, what was that like?
- What is your attitude like towards people with schizophrenia?
- Has this changed at all since you started work?

1. **What was it like starting the community engagement work?**

**Prompts**

What are the positive aspects of this work so far?

What are the negative aspects?

Did you experience and resistance or difficulties? Tell me about those

1. **How supported do you feel in delivering CBR?**

**Prompts**

- How do you feel about your personal safety?
- How accessible is your supervisor?
- How useful are your supervision meetings? How might they be made more useful for you?

**FOR CBR SUPERVISORS:**

1. **What was it like supervising CBR?**

**Prompts**

- What is your relationship like with the CBR worker?
- What was it like working with a person with schizophrenia +/- intellectual disability?
- How confident do you feel in supervising CBR?
- How acceptable do you think it is to ask about suicide risk and chaining at every home visit?
- What was your role in community engagement work?

1. **What were your experiences of managing difficult situations?**

**Prompts**

- How do you feel about the instructions/ flow charts?
- How do you feel about the support you got for managing these situations?
- What was your experience of adverse event reporting, if this occurred?

1. **How supported do you feel in supervising CBR?**

**Prompts**

- How do you feel about your personal safety?
- How accessible is the trial coordinator/ trial manager?

**RISE pilot initial evaluation (2 months): Topic guide for IDIs with health centre staff**

1. **What has been your experience of having contact with CBR workers so far?**
2. **What was it like when the CBR worker came to the health centre with the patient?**

**Prompts**

- Did the presence of the CBR worker affect your work in any way?
- How does CBR affect the care that you can give the patient? E.g. do they help or hinder the care you provide?
- How does CBR affect your relationship with the patient and caregiver?
- What is your relationship like with the CBR worker?

1. **How useful do you think CBR is for the participants?**

**Prompts**

- How has CBR impacted upon the individual’s understanding about schizophrenia?
- How has CBR impacted upon the individuals’ ability to access care?
- How has CBR impacted upon human rights issues such as chaining?
- Do you notice any impact of CBR even when the CBR worker is not there?

1. **What challenges do you have relating to the CBR workers?**

**Prompts**

- Are there any difficulties you faced relating to the CBR workers?
- How did you overcome these challenges?
- Is there anything that can be done to make it easier for you to work alongside CBR workers?

**Community leaders pilot end line**

1. **Are you aware of any people with mental illness living in your area?**
2. **Whose responsibility do you think it is to support people with mental illness?**
3. **Have you been involved in supporting people with mental illness in anyway?**

Please tell me about how you have been involved.

If they haven’t been involved before ask: How might you be involved in the future?

*[Orientate the participant to what we mean by CBR. Distinguish from PRIME (care at the health centre) and health extension workers]*

1. **If the participant has been involved: How has the CBR worker affected your own involvement?**

**Prompts**

- Were you aware of the person/people with mental illness before the CBR worker started?
- In what way were you involved in supporting the person before the CBR worker started?
- How has the CBR worker increased or decreased your involvement?

1. **If the participant has been involved: How do you feel about being involved in supporting the person with mental illness?**

**What difficulties, if any, did you have in supporting the person?**

**Prompts**

- Do you feel happy to help?
- Has it taken your time?
- Have you used your own funds?
- Have you had any stigma towards yourself for giving this support?

1. **In what circumstances do you think the CBR should ask community leaders/ members to help support a person with mental illness?**

**Prompts**

- Is it appropriate in all cases or just some cases?
- What kind of support should the CBR worker look for from community leaders/ member?
- Financial support?
- Paying for medication?
- Fetching medication?
- Food?
- Housing?
- Mediation/ discussion with the family?
- Finding employment?
- Encouragement to the individual?

1. **How important or useful is support from community leaders/ members for people with mental illness?**
2. **In what ways do you think CBR has helped the person with mental illness?**

**Did it help with…**

…independence?

… self care?

…doing household tasks?

…symptoms?

…accessing health services?

…taking medication?

…social and community life? For example, participating in Edir, going to church, drinking coffee with others..

…getting back to work?

...the family environment?

…physical health?

…substance abuse?

…stress and anger?

…experiences of stigma and discrimination?

…self esteem?

…caregiver burden?

…physical restraint?

…managing a crisis/ relapse?

1. **In what ways has CBR changed awareness about mental illness in your community?**

- Have attitudes changed?
- Has knowledge changed?
- How has this impacted on individuals with mental illness?

1. **In what ways can CBR be improved?**

In particular how can the involvement of community leaders/members be improved?

**RISE Pilot qualitative endline interviews with participants**

1. **How did you feel about receiving CBR?**

If you weren’t happy to receive CBR please tell me why not (e.g. didn’t find it useful)

1. **How has CBR impacted on your life?**

**Prompts**

**Has it impacted on any of the following areas?**

…independence?

… self care?

…doing household tasks?

…symptoms?

…accessing health services?

…taking medication?

…social and community life? For example, participating in Edir, going to church, drinking coffee with others..

…getting back to work?

...the family environment?

…physical health?

…substance abuse?

…stress and anger?

…experiences of stigma and discrimination?

…self esteem?

…caregiver burden?

…physical restraint?

…managing a crisis/ relapse?

1. **What was the impact of CBR on your taking medication or not?**

**Did CBR help with any issues with:**

- Medication availability?
- Medication cost?
- Side effects?
- Remembering to take it?
- Being told **not** to take it by others (e.g. relatives, holy water priest)

1. **Were there any negative impacts of receiving CBR?**

- Decreased time for other activities e.g. farming
- increased stigma from home visits

1. **What was the role of the CBR worker in any changes over the last year?**

In particular did any of the following help?

- Having home visits from the CBR worker
- Having CBR worker accompany or remind you to go to the health centre?
- Having someone you can trust to talk to
- Family support group [if applicable]
- The CBR worker giving education to the community and making links in the community

1. **Aside from CBR, what other factors have helped to improve your situation?**

**Prompts**

- Having support, e.g. from family, support from community worker, support from community
- Finding work, getting financial support
- Taking medication regularly; getting free medication
- Community having increased awareness
- Spirituality/ prayer/ help from God, holy water,
- Accepting circumstances, understanding illness/knowing what to expect,
- Improved self-esteem/ positive attitude,

1. **Did anything make it difficult to participate in CBR?**

**Prompts**

- not having enough time,
- increased stigma from home visits

1. **What could have made CBR more helpful for you?**

What would you like to see changed about the CBR programme?

How useful do you think the CBR programme might be for other people in your situation?

1. **Are you aware of any work the CBR worker did in your kebele, aside from visiting you at home?** e.g. community awareness raising meetings, meetings with businessmen/ priest with the aim of supporting you etc

**If yes🡪 Please tell me what you think about this community work**

**Prompts**

Do you think this community work is useful?

Did this community work impact upon you in anyway?

Did this community work have any negative impacts on you?

1. **How do you feel about the fact that CBR is finishing?**

Do you feel that CBR has given you skills/ knowledge that will continue to help you even though the CBR worker will stop coming?

For caregivers: **how confident/ able do you feel to look after you relative?**

**RISE pilot endline: Topic guide for IDIs with CBR supervisors/ FGDs with CBR workers**

1. **What is your experience of being a CBR worker/supervisor – positive or negative**
2. **How useful do you think CBR is for the participants?**

**Prompts**

- - What expectations do you think the individuals and families had of CBR?
  - Was the CBR worker able to meet those expectations? Did participants eventually understand that CBR would not be able to provide financial support or free medication?
  - In what ways do you think home visits are useful? Give me some examples from your experience.

1. **What is the most important ingredient of CBR in your opinion?** E.g. just having the home visits/ contact with the CBR worker? Any specific module? The community work?
2. **In the last 12 months have any changes occurred relating to the following** *[for each probe follow up with ‘Tell me about the change’ AND ‘What was the role of CBR in the change?’]:*

…independence?

… self care?

…doing household tasks?

…symptoms?

…accessing health services?

…taking medication?

…social and community life? For example, participating in Edir, going to church, drinking coffee with others..

…getting back to work?

...the family environment?

…physical health?

…substance abuse?

…stress and anger?

…experiences of stigma and discrimination?

…self esteem?

…caregiver burden?

…physical restraint?

…managing a crisis/ relapse?

- **In your opinion on which aspect of life does CBR have the greatest impact?**
- **Did CBR have the impact you expected on the participants? Were you surprised by anything? Were you disappointed by anything?**

1. **What other factors, apart from just CBR, do you think had an impact on the individual**

**Prompts**

- Having support, e.g. from family, support from community worker, support from community
- Finding work, getting financial support
- Taking medication regularly; getting free medication
- Community having increased awareness
- Spirituality/ prayer/ help from God, holy water,
- Accepting circumstances, understanding illness/knowing what to expect,
- Improved self-esteem/ positive attitude

1. **What do you think will happen to the individual/ family you were working with after CBR is finished?**

- Do you think any positive effects of CBR will continue? Tell me more about this...
- What is the role of the caregiver in continuing positive effects of CBR?
- What is the role of the community in continuing positive effects of CBR?

1. **Tell me about your experience of the community engagement work**

**Prompts**

- What are the positive aspects of this work so far?
- What are the negative aspects?
- Did the CBR worker experience and resistance or difficulties? Tell me about those
- How much does community engagement work (including awareness raising, individual meetings with community leaders etc) have an **impact** on people with schizophrenia?
- In the manual and training there is the task to ‘demonstrate the progress of people with schizophrenia to the community’. There wasn’t a chance to do this in the pilot. Why do you think this wasn’t possible? Can you imagine a situation where it would be possible? If not, why not? What impact do you think this could have if it was possible?
- What are the particular issues with arranging employment opportunities for people with schizophrenia? How useful is this part of CBR?
- Why was there no involvement with traditional healers- is it taboo, not relevant to participants, are they hidden?

1. **What challenges did you have in your work?**

**Prompts**

- Was there anything that made it difficult for individuals and families to participate?
- What were the reasons why people stopped CBR?
- Was there anything that made it difficult for you to supervise/deliver CBR?
- How did you overcome these challenges?
- Is there anything that can be done to make it easier for you to supervise CBR?
- Is there anything that can be done to make it easier for CBR workers to deliver CBR?
- Is there anything that can be done to make it easier for individuals and families to participate?
- Would you like to continue supervising CBR work?

**FOR CBR WORKERS:**

1. **What was it like doing CBR home visits?**

**Prompts**

- What is your relationship with the individual and family like? How has this changed over time?
- How confident do you feel in delivering CBR? How has this changed over time?
- If you had to deal with any difficult situations, what was that like?
- What is your attitude like towards people with schizophrenia?

How has this changed over time?

1. **How supported do you feel in delivering CBR?**

**Prompts**

- How do you feel about your personal safety?
- How do you feel about the emotional burden on you?
- How accessible is your supervisor?
- How useful are your supervision meetings? How might they be made more useful for you?
- How useful are the group supervision meetings? Do you also discuss CBR with other CBR workers outside of these meetings?
- How do you deal with stress?
- How do you feel about the amount of top up training you received? How useful was it?

**FOR CBR SUPERVISORS:**

1. **What was it like supervising CBR?**

**Prompts**

- How is your relationship with the CBR worker? **How has it changed over time?**
- What was it like working with a person with schizophrenia +/- intellectual disability?
- How confident do you feel in supervising CBR? **How has this changed over time?**

1. **What were your experiences of managing difficult situations?**

**Prompts**

- How do you feel about the instructions/ flow charts?
- How do you feel about the support you got for managing these situations?
- What was your experience of adverse event reporting, if this occurred?
- Have your experiences or feelings changed over time in relation to managing difficult situations?

1. **How supported do you feel in supervising CBR?**

**Prompts**

- How do you feel about your personal safety?
- How accessible is the trial coordinator/ trial manager?
- How do you feel about the emotional burden on you?
- Do you ever feel stressed? How do you deal with stress
