# Supplementary material for: Community-based rehabilitation intervention for people with schizophrenia in Ethiopia (RISE): a 12 month mixed methods pilot study
Source: BMC Psychiatry. 2018 Aug 3;18:250. doi: 10.1186/s12888-018-1818-4 (PMC6091097; doi:10.1186/s12888-018-1818-4)
Supplement: Supplementary file 5 — Instruments. Word document. Further details on quantitative data collection instruments. (DOCX 21 kb) [file 12888_2018_1818_MOESM5_ESM.docx]

| **Outcome** | **Instrument/s** |
| --- | --- |
| **Lay data collector: patient-reported** | |
| **Discrimination** | Measured using Section 1 of the Discrimination and Stigma Scale-12 (DISC-12) [13]. Original version includes 21 items asking the participant if they have experienced different types of discrimination relating to their mental illness. All items are rated on a 4-point likert scale (0 ‘Not at all’ to 3 ‘A lot’). Due to PRIME’s initial adaptation of DISC-12 for Ethiopia, at baseline the DISC-12 did not include items relating to ‘unfair treatment in accessing welfare benefits’ and ‘privacy’. These items were introduced at midline and endline but these items were excluded from summary scores at all time points to allow time points to be compared. |
| **Alcohol use** | The Alcohol Use Disorders Identification Test (AUDIT) is a 10-item tool, which will be used to detect hazardous drinking [16]. Possible score range 0-40 with higher scores indicating greater severity. |
| **Depression** | The Patient Health Questionnaire- 9 (PHQ-9) [17, 18]. PHQ-9 incorporates DSM-IV depression diagnostic criteria with other leading major depressive symptoms into a brief self-report tool. Each item rated as ‘0’ (not at all) to ‘3’ (nearly every day). The item ‘Feeling bad about yourself’ was excluded at baseline as part of the initial adaptation for Ethiopia by PRIME, but reintroduced at midline at endline. This item was excluded from summary scores at all time points to allow time points to be compared. Possible range for adapted version is 0 to 24 with higher scores indicated greater severity. |
| **Lay data collector: caregiver-reported** | |
| **Disability** | 36-item WHODAS 2.0 [9]. 6 domains: understanding and communication, getting around, self-care, getting along with people, life activities and participation in society. Total scores range from 0-100 with higher scores indicating higher levels of disability. At baseline data collection, both people with schizophrenia and caregivers contributed to the WHODAS responses, due to different procedures utilised by PRIME at that time point. At midline and endline only caregiver responses were used. |
| **Caregiver burden** | Involvement Evaluation Questionnaire (IEQ) [19]. This is a 31-item questionnaire assessing aspects of caregiver including tension, worrying, urging and supervision. All items are scored on 5-point Likert scales (0 never to 4 always). A 27-item sumscore can be computed (range 0 to 108). |
| **Psychiatric nurse-rated instrument** | |
| **Clinical impression** | Clinical Global Impression (CGI)- illness severity [12]. Rated on a seven point score ranging from 1, when the patient is assessed to have no illness, to 7, when the patient is among the most severely ill. The scale is completed entirely based on clinical judgment. |
