# Supplementary material for: Community-based rehabilitation intervention for people with schizophrenia in Ethiopia (RISE): a 12 month mixed methods pilot study
Source: BMC Psychiatry. 2018 Aug 3;18:250. doi: 10.1186/s12888-018-1818-4 (PMC6091097; doi:10.1186/s12888-018-1818-4)
Supplement: Supplementary file 6 — Assumptions table. Word document. Summary of main findings, adjustments to intervention and conclusion relating to each theory of change assumption. (DOCX 20 kb) [file 12888_2018_1818_MOESM6_ESM.docx]

| **Assumption** | **Main findings** | **Adjustments to intervention** | **Conclusion** |
| --- | --- | --- | --- |
| **Research question 1: Is the RISE CBR intervention acceptable?** | | | |
| **1a: People with schizophrenia and caregivers are willing and have time to participate in CBR** | **Process:** Three participants dropped out before 12 months (1 x 8 months, 2 x 11 months). Mean home visits 21 (range 17-27) **Qualitative:** Most welcomed CBR; Close relationships often formed with CBR workers. Disengagement due to improved functioning, disagreements over medication use, and participants visiting holy water/ relatives. Some issues about home visits interfering with work- for men. Some issues with engaging suitable caregiver. Some participants wanted written information. Community mobilization generally acceptable. | Extend Phase 1 to 3 months. Adjust frequency, duration & location of visits to fit needs of participants. Maintain phone contact between visits. Continue CBR even if no engagement from caregiver. Provide written materials for CBR participants. Top up training on recovery model- balance between encouraging medication and accepting can not or will not take it. Clarify that participants can continue CBR despite not taking medication. | Assumption shown to be correct and converted to rationale, as majority received adequate CBR. Monitor in trial (process and qualitative) as increased flexibility may be more difficult at scale. |
| **1b: CBR can meet the needs of people with schizophrenia** | **Process:** All participants achieved all core goals. Between 6-11 optional goals selected, of which between 1-8 achieved. Tendency to select too many goals, not achievable in time frame. Some caregivers disappointed in slow progress, especially if comorbid intellectual disability.  **Qualitative:** Participants often wanted to resume gendered family role. Poverty was a major issue which CBR could not directly address- leading to some disengagement with CBR. | Better explanation on lack of financial support. Add extra potential goal ‘Person with schizophrenia can fulfill family role'. Top up training to ensure focus on fewer more achievable goals. Focus on maintaining goals in phase 3, rather than starting several new ones. *Trial evaluation:* Add focused qualitative interviews around recovery. Add endline process assessment on whether CBR met need. | Keep as assumption as unresolved issues around impact of CBR in particular the ability of CBR to alleviate poverty (quantitative and qualitative) |
| **1c: CBR workers are willing to work with people with schizophrenia** | **Process:** No CBR worker withdrawals. **Qualitative:** Attitudes changed over time (concerns about violence more realistic; increased expectation of recovery). Group supervision helpful. |  | Assumption shown to be correct and converted to rationale. |
| **1d: Community leaders willing to support CBR without benefits for themselves** | **Process:** Awareness raising and meetings with community leaders conducted in all sub-districts. Targeted mobilisation of community leaders attempted for six participants.  **Qualitative:** Initial difficulty arranging meetings. Some community leaders implied per diem needed for meetings exclusively for CBR. Otherwise community figures happy to participate. | Only do community meetings attached to another planned meeting- better attendance and minimize expectation of per diem. For individual engagement target those unlikely to ask for personal benefit. | Assumption shown to be correct and converted to rationale. Monitor at scale in trial (qualitative and process) |
| **1e: Traditional and religious healers willing to support CBR** | **Process:** No CBR engagement with traditional healers- holy water sites out of district. |  | Keep as assumption and monitor in trial (qualitative and process) |

| **Research question 2: Is the RISE CBR intervention feasible?** | | | |
| --- | --- | --- | --- |
| **2a: Non specialists can be trained to deliver CBR for people with schizophrenia** | **Process:** All CBR workers retained. Some variation in abilities. Areas needing additional training: risk assessment, physical health/substance use assessment; problem solving, eliciting feedback, normalization of feelings, coping mechanisms; comorbid intellectual disability. Supervision frequency less than anticipated (mean 10 individual sessions, 8 group sessions, 5.4 unannounced visits) **Qualitative:** Participants happy with manner & knowledge of CBR workers. CBR workers and supervisors mostly happy with training and support; sometimes slow response to difficult situations. | Top up training given on weak areas and how to support people with comorbid intellectual disability. Produced more compact step-by-step guide with difficult situation flow charts, added details about side effects. Improved forms- made easier to use.  Keep supervision expectations the same. | Assumption shown to be correct and converted to rationale. |
| **2b: CBR workers can overcome logistical challenges to deliver CBR** | **Process and qualitative:** Time consuming to access some households due to long distances and shortage of public transport. Difficulty arranging visits/ supervision due to telephone network problems. Importance of home visits to ensure engagement and understand family environment. | Increase transport allowance for CBR workers and increase access to project vehicle for supervisors. Reduce maximum number of participants per CBR worker for trial. | Keep as assumption. Need to monitor in trial as greater number participants per CBR worker and longer distances. |
| **2c: Primary care staff are supportive of CBR** | **Process:** Accompany to health centre twice on average over 12m. 14 referrals to health centre for 8 participants.  **Qualitative:** Health officers good relationship with CBR workers once understand each roles. Feel CBR is beneficial. | At project start give orientation to CBR project and CBR workers, with clearer explanation of expectations. | Assumption shown to be correct and converted to rationale. |
| **2d: Anti-psychotic medication is accessible** | **Process:** Medication supply issues at health centre; can't prescribe without receipt. **Qualitative:** Difficulties affording medication. Attempts to access free medication certificate unsuccessful. Many problems with side effects, commonly reported weakness which caused problems with doing farm work which needed to survive. Some issues with not taking alongside holy water. | Gain better understanding of how to access medication fee waiver. No plan to pursue free medication provided by CBR as this would not be sustainable or scalable.  *Trial evaluation:*  Additional medication adherence questions to assess affordability and availability | Keep as assumption and monitor in trial (qualitative, process and quantitative) |
| **2e: Edir support will be available and sustainable** | **Process:** Edir groups used for awareness raising but no financial support. Groups and some CBR participants not interested. **Qualitative:** Individual businessmen used as benefactors in urban area. Some doubts about sustainability. | Edir support not included as component; focusing efforts on individuals may be more successful. | Remove assumption. Amend community support to include businessmen. |

| **Research question 3: Can the RISE CBR intervention have an impact and if so, how?** | | | |
| --- | --- | --- | --- |
| **3a: CBR can improve functioning in people with schizophrenia** | **Quantitative:** Substantial improvements in disability, symptoms, alcohol use, depression, discrimination and caregiver burden over the pilot period.  **Qualitative:** Increased understanding about mental illness preceded increased family support and access to healthcare. This led to increased functioning by improving symptoms, decreasing stigma/abuse and social inclusion and increasing income. Improved livelihood most important outcome for many. Increased hope and self-esteem underpinned and sustained functioning progress. Overly ambitious goals in some cases. | No major adjustments. CBR worker refresher training on focusing on fewer more achievable goals; striking balance between realism and hope. | Keep as assumption. Monitor in trial (quantitative) as randomized evaluation required |
| **3b: A community mobilisation approach is required in addition to home-based care** | **Qualitative:** Improved attitudes from awareness-raising led in some cases to signposting other people with schizophrenia to health centre. Tangible support facilitated for 4 participants (medication, housing, moral/spiritual support, food). Financial support from community was only available in urban area for people with schizophrenia with obvious needs. | No major adjustments. | Assumption shown to be correct and converted to rationale. Monitor specific aspects in trial (qualitative and process) |
| **3c: Family support groups are perceived to be useful despite not having savings and loans element** | **Process:** 1 group set up- 3 meetings. **Qualitative:** One woman with schizophrenia found it relieved stress. Groups not set up as too few participants; did not want to discuss problems; too ill to attend. | Family support groups were designated as an optional CBR component depending on the wishes of participants in each sub-district. | Keep as assumption and monitor in trial (qualitative and process) |
