# Supplementary material for: Community-based rehabilitation intervention for people with schizophrenia in Ethiopia (RISE): a 12 month mixed methods pilot study
Source: BMC Psychiatry. 2018 Aug 3;18:250. doi: 10.1186/s12888-018-1818-4 (PMC6091097; doi:10.1186/s12888-018-1818-4)
Supplement: Supplementary file 7 — Supplementary qualitative data. Word document. Additional supporting quotations from qualitative data, by theme and sub-theme. (DOCX 42 kb) [file 12888_2018_1818_MOESM7_ESM.docx]

**Supporting qualitative data**

## Research question 1: Is the RISE CBR intervention acceptable?

### Assumption 1a: People with schizophrenia and caregivers are willing and have time to participate in CBR

*“[The CBRW] told us that we can count on him and call him when we experience some problems. He told us that he will try to solve the problem by cooperating with us […] Other individuals might not keep the secret confidentially. For example, if they heard that he has mental illness, they may say that he is “crazy” [...] It is better to tell [the CBRW] and get advice from him. This is because he is a teacher and will keep the secret confidential.”*

(female caregiver-7-E)

*“[The caregiver] received us warmly. I also had a very good relationship with both of them. We had a very good time. We were like friends […] I regret that I didn’t have pictures taken with [the CBR participant] before he left for Addis for work.”*

(male CBR worker -8-E)

*“it is because until God heals her from this illness, we are happy to get the education”*

(female caregiver-10-B)

*“There was no anytime which they had resisted the service. They wait for me being at the garden with chairs for our appointment.”*

(CBRW-B)

*“I: Have you worried lest people should know that she is coming to your house because she is ill? R: Why should I worry? The lesson is for my own good. It doesn’t worry me. We get benefit from the education and we have nothing to lose”*

(female caregiver-10-E)

*“I: Did your Community Based Rehabilitation education cause any problem? R: No. I: For example, you may have works to be done or social affairs to be participated during the Community Based Rehabilitation education time. R: Yes, you are right. I: What did you do at that time? R: This is the health of my son; I prefer to save him than anything. I: Didn’t the education take your time? R: Yes, it takes. I: Don’t you think it is a problem? R: It will not be a problem. He will consult me when to make the education and we will arrange the appropriate time together. I: Did you face any stigma when your neighbors see the trainer with you? R: No. I: Didn’t you feel shame? R: There is nothing here that feels me shame. Instead I would be very happy.”… I: Did you fear gossips from residents in the near while they know the reason why the Community Based Rehabilitation worker is in your home? R: I don’t care for whatever they said as long as my son is cured.”*

(female caregiver-8-E)

“I: What about the time he takes to you for the CBR? Does he takes your time or is it not convenient for you? P: I used to do my handicraft work while I talk to him. ...yes... I didn’t have any problem with it as I could do my work while talking with him.”

(woman with schizophrenia-4-E)

“I: Didn’t her presence created inconvenience?

P: No, she didn’t. ...eh... She even let us to do the work instead.

I: Is that?

P: Yes.

I: Okay.

P: She even came when I was producing local alcohol. ...eh... She respects our work. .... eh... the work ...”

(woman with schizophrenia-10-E)

“Interviewer: Was [the CBR worker] the kind of person you can put your trust in?

Respondent: Yes. We have become family. She is from this area. She knows many things about us. It is difficult for a person who lives far away [from us] to understand our situation.”

(female caregiver-10-E)

“[The CBR worker] reminded me to take the medication properly, but I was not happy with her reminder… I thought she considered me as a child. However I understood that it is helpful”

(man with schizophrenia -1-B)

“I: Did the CBR education he was giving you consume your time? R: Yes. The lesson was very good. But I also have to craft the glass, frame it and deliver it to my customers. You can think of how much time it took us to finish our interview now. Isn’t it a very long time? I: Yes. R: My customers will start saying that I am arrogant when I fail to supply their order on time.”

(man with schizophrenia-6-E)

“I didn’t return [the CBR worker’s] calls because I thought ‘I have recovered from my illness’. I have also completed the lesson that was planned for one year […] I have recovered because of the medication that was prescribed. There are doctors here. I can go to them, if my illness relapses.”

(man with schizophrenia -6-E)

“When people ask me why [CBR workers] are coming to my house, I tell them that I am a member of ihadig [the ruling political party] and that I am being interviewed. They will be afraid.”

(man with schizophrenia -6-E)

“I should know these things [about mental illness]. I don’t care if the society knows [this information] or not. If you teach elders, when something happens ... in my family, they will come to negotiate [with] us. At that time when I talk loudly they will say, “you are mad”. I am not happy with them being taught.”

(man with schizophrenia -8-E)

“Is there anything which doesn’t make you happy or not interested about? P: There is nothing which disappointed me. …eh… I don’t want too much question. I don’t feel good. …eh… I prefer to be asked brief and few questions. …eh… I also want to answer those questions. I: Do you want that? P: Yes. I: Did she ask you long questions? P: Yes.”

(man with schizophrenia-1-E)

### Assumption 1b: CBR Can address the needs of people with schizophrenia

*“I: What should she improve in her teaching?*

*R: What could I say? She is a trained worker … I simply accepted what she told me.”* (woman with schizophrenia -10-B)

*“We didn’t expect perfection. But we thought they were capable of implementing what we taught them. [Before CBR] they were not aware that they had to take the medication for their illness. We thought they would take the medication, if we had taught them... But they may not be able to take the medication, even if they wanted to, if they don’t have money.”*

(female supervisor-E)

*“When I first saw [the CBR*W*], I thought as my daughter [has] got mercy from God. That was my first impression.”*

*(*Female caregiver-2-B*).*

*“The [CBR participant] directly told us that she won’t continue CBR unless [financial] aid is given to her … She was saying that her child is sick and we are only teaching about medication and telling her to bring medication. She was complaining [that we were] not doing anything for her.”*

(CBRW-4-B)

*“It is more understandable to read by myself than hearing what they are teaching”* (female caregiver-5-B)

### Assumption 1c: CBRWs are willing to work with people with schizophrenia

*“Throughout our work we are communicating as good friends. …eh… That aggressiveness, that e… hostility e… loneliness has changed with friendship. When this has been improved, all things are changing.”*

(female CBRW-9-E)

### Assumption 1d: Community leaders are willing to support CBR without benefits for themselves

*“It took [the community leaders] a long time to accept what we were doing. We had a very big challenge to do the work because it was election time. We made the [sub-district] chairman participate in our discussion and he approved of the work we were doing and gave us a letter of permission. Then we started doing the work after two months.”*

(male CBRW-8-E)

*“Some of them because they don't know [about mental illness].. some ask why I talk with [the man with schizophrenia], “he is crazy, he has lost his mind why would you talk to him?” When they say such kind of things, [I say] no this is not right. We should show them love, include them in any society, include them in social activities.”*

(priest-5-E)

*“You feel happy when you help someone whose economy is below you. When you see improvement in the person you are helping, you will be satisfied. I am very glad since I have helped him. I got happiness”*

(male benefactor-7-E)

*“As they [the community leaders] think the [RISE] project as an ngo, they expect some benefits. At this time it could be the awareness problem. It is also not understanding the benefit of the CBR service delivery in that sub-district. After telling them about the service they think as we deliberately avoiding them from the benefits. ”*

(female CBRW-9-E)

*“Some of them don’t want to participate because they have other things to do. … But they want to participate in the discussion after they see benefits. After we gave them awareness, they were telling us to teach them in the meetings.”*

(male CBRW-11-E)

## Research question 2: Is the RISE CBR intervention feasible?

### Assumption 2a: Non specialists can be trained to deliver CBR for schizophrenia

***“****The CBRW has both the ability to understand and explain issues … he both explains in a way which is understandable to us and listens and understands what we tell to him … we even ask him [that] which is not clear to us..”*

(man with schizophrenia-6-B)

*“[the CBR worker] is so brilliant, she has good experience and she gives good care, I would be happy if this work hadn’t stopped and she continued her job with us”*

(male caregiver-7-E)

*“The education [CBR] didn’t lack anything. They were delivering the education in a very good way. I am happy about that.”*

(man with schizophrenia-5-E)

*“I don’t have any complain about her lesson. What makes give up hope is that he thinks the medication is hurting him and wants to interrupt taking it. We always fight over this issue. When she was coming to our house, he never wanted to listen to her advice, even for thirty minutes. He thought that we were nagging him. I get very much upset, when he says that her lesson is not important to him.”*

(female caregiver-1-E)

### Assumption 2b: CBRWs can overcome logistical issues to deliver CBR

*“We could get the patient and the caregiver only if we appoint them somewhere. However at home we could get all the family and even the neighbours. So that we could change attitude of the community. …eh… If we change the attitude of one caregiver, it might not have an impact as the caregiver mightn’t be changed. …eh… However if it is at home, we could discuss with the whole family. That could fasten their improvement.”*

(CBRW-B)

*“The other benefit which the home visit have is the value which the patient feels as somebody is going to his home for visit. …eh… The patients could have good feeling that people are concerned about their health.”*

(CBRW-B)

*“I am also happy when they visit coming to my house. …eh… I feels honoured.”*

(man with schizophrenia-1-B)

*“there are remote areas where the t workers are expected to visit. I think these remote areas should be identified. For example if we expect a CBR worker to go to a place that he would have to go through a forest full of snakes. If we expect the CBR worker to go to such kind of places every week that might be a bit difficult. These is because some day he might be harmed. In my opinion I don't think that we should work in such kind of areas, I don't want to see any of our colleagues get hurt. These could also affect me because I would be required to go to a place where the CBR worker visits.”*

(male supervisor-E)

*“Their house is the best place to meet [CBR participants]. You can observe their family relationships, if you go to their house. You can give them practical advice, if you know how they are living. Going to their houses is also very useful to meet their neighbors and do community work.”*

(female supervisor-E)

### Assumption 2c: Primary care staff are supportive of CBR

***“****There is no negative influence, as I have told you before. They do as we tell them to do. They follow our advices. We can protect patients’ privacy by telling them (The CBR workers) and even patients’ families to stay away from us when we want to talk to the patients in person.”*

*(Health officer)*

### Assumption 2d: Anti-psychotic medication is accessible

*“The tablet they were giving me…made me clumsy and unable to work. It makes you drowsy and you will doze off in your seat. If you take it at night, it will make you sleep until noon. Do you think my wife will stay with me if I can’t provide?”*

(man with schizophrenia-6-E)

*“[A relative] asked us why are we urging [his sister] to take medication. He thought that we have an interest to kill her. He told [me] that the medication had created problems for her health”*

(male CBRW-3-B)

*“Therefore we were in bad situation as they were refusing the CBR service and didn’t want our visit. However we agreed with them to continue the CBR though the medication has stopped. They were thinking that the treatment was the cause for her disability. They said that she was capable of performing activities at home but the medication made her lay on bed. Though the problem with the medication couldn’t be solved, the CBR has continued to be delivered.”*

(male CBRW-3-E)

*“The kebele, the community, the mentally ill people, and their families have a problem regarding the selection of the poorest of the poor for free access to treatment and medication. They select these people using their own criteria and without discussing the issue with us. They have confusion about who needs to get help. The kebele [management] says people who have land can sell their land and get treatment. But other people say that they have to be helped because they have a mentally ill person in their house who is not able to work and support himself. This was the problem I failed to solve in the pilot program. The supervisor has also tried his best to make her accept the situation, but she couldn’t. I think that is the very reason she didn’t want to continue participating in the CBR”*

(male CBRW-4-E)

*“The kebele mightn’t cooperate to give the support letter for free medication. They are not willing to cooperate. …eh… They don’t want to give for anyone who went there. They consider those who are homeless as those who need free medication.”* (CBRW-E)

## Research question 3: Can the RISE CBR intervention produce an impact and if so, how?

**Assumption 3a: CBR can improve functioning in people with schizophrenia**

*“Previously [the CBR participant] even didn’t breastfeed her baby, instead she viewed him as a wild beast. …Now she is caring by feeding him properly. In the past she wasn’t able to prepare food. When we went to her home, we found her asleep. ..There were so many problems related to her life. Now she got all these improvements of feeding her children properly and doing household activities.”*

(male CBRW-11-B)

*“I used to grow my beard for more than one year and my hair was infested with pests, because I didn’t wash it for four or five months…I wasn’t changing my clothes. But now I have started washing my clothes and my hair. The training has helped me to become a better person.”*

(man with schizophrenia -5-E)

*"for the positive impact [of CBR] to continue for the future, I don’t think it to continue. It is because the gaps are visible. …eh… As the time we get in to their home is becoming long, the gap will be wider. …eh… The care they give to her when I went there is different from when I am not there. When I go there they care for her too much but when I went there accidentally, it was not good. Though they have focus on the care, I think this will continue with minimal commitment.”*

(female CBRW-2-E)

*“There is a huge disagreement between family members and that has an impact on the mentally ill patient. It is the problem within the family that makes the patient to be disturbed, angry, go out of the house, avoid themselves, not care for themselves, not eat food and not keep their hygiene.”*

(male supervisor-E)

*“We know when the illness is about to relapse. She will start getting upset easily and she will also start talking to herself. We will give her the medication, if we have it at home. Or, we will go out to the health center to bring the medication […] Her lesson has changed us a lot. It is a big deal for us, to know where to take her [when her illness relapses].”*

(caregiver-10-E)

***“****This person is sick and we prepare how to pass the time of the illness. We knew how to manage the relapse. I know my husband’s behaviour more than anyone.… When he feels angry, I pass that with a smile. I also take children away from him.”* (female caregiver-1-B)

*“most of [the CBR participants] didn’t have a belief that schizophrenia could be treated through modern treatment … There was no habit to go to the health centre. … Even those who went there interrupted it.… However now as they got awareness from the CBR, they follow their appointment properly. Therefore they now understood that the treatment has important impact on their health.”*

(male supervisor-B)

*“[the CBRW] has told me to go outside the house when I and my wife start fighting. But she has almost stopped nagging me after his advice. I also go outside, when I see changes in her face. Both of us calm down when I get back home.”*

(man with schizophrenia -6-E)

*“They told me [the man with schizophrenia] was tied up because he was fighting with [the caregiver] over taking some household property outside the house […] They tied [the man with schizophrenia] up on Monday night. I went there at the appointment time on Tuesday. So, I met [the caregiver], talked to her and listened about him. We took [the man with schizophrenia] to the appropriate place and he was released. So, [CBR] visits are very important. The burden she was carrying was heavy. We told her that he was showing the symptoms of mental illness and that he will get better if he is taken to the hospital and takes his medication properly and had him released.”*

(male CBR worker -8-E)

*“[the CBRW] tells me I will get better..She motivates me to be strong… If she didn’t come I would be ill I would be in bed…If she wouldn’t come who would teach me this whole thing? …She makes me feel better”*

(woman with schizophrenia -9-E)

*“What was amazing was, [the CBR participant] was not attending wedding ceremonies, or mahber [social religious meetings]. She was also not going to church. Now she has started going to church. I have seen her at the church on Christmas and Epiphany. This is a pleasant change.”*

(female CBRW-10-E)

*“I benefited from [the CBR* *worker’s] education […] The CBR worker taught me that whatever someone says to me, I don’t have to respond to him. Arguments will affect my health condition****.”***

(man with schizophrenia -8-E)

*“R: [The illness symptoms] decrease when they take the medication properly. They will gradually recover from their illnesses. There was this girl who used to rove the streets without any rest. She became stable and started settling in the house when she started taking the medication properly… Of course, the [medication] will make them drowsy, but their habit of speaking to themselves will reduce. It will relapse when they interrupt the medication.”*

(female supervisor-E)

*“We were waiting for the medication for her mental illness. When it comes here in the health centre, I became happy because we got relief as she had no sleep and disturbs us. We lost sleep for long period of time. She shouts and talks throughout the night. We had much stress because of that. …eh… Now thanks for God, she has improved from her previous situation. We have found improvement in her health.”* (female caregiver-10-B)

*“We told her to tell them that she needs rest in between and continue her work after she had her lunch. …After her improvement she is an active participant in household tasks such as fetching water early in the morning….If she is baking enjera the others will do the other work. This agreement is an improvement.”* (female CBRW-9-E)

*“People advise me to go to Ammanuel hospital or to holy water. I didn’t accept that. I want to go when I need by myself. I don’t want to be pressurized by anybody. I: Did the stigma minimize after the CBR? R: Yes, it decreased a lot. I benefited from [the CBRW’s] education. The CBR worker taught me that whatever someone says to me, I don’t have to respond to him. Arguments will affect my health condition.”* (man with schizophrenia-8-E)

*I have started doing some work. I used to ruin my house and sleep somewhere else. Now, I have repaired my home and have fenced the compound. What I am left with is getting married. People might recall the wrong things I did to my ex-wife…. But God has cured me of my [mental] illness now.*

(man with schizophrenia -5-E)

### Assumption 3b: A community mobilisation approach is needed in addition to home-based care

*“When he was ill in the past, he used to get drunk and speak louder. The people in the neighbourhood heard him and they used to care and feel pity for me. They understood that taking care of a patient is very difficult. So if the people had the awareness about the illness and understood the symptoms of the illness, they will help.”*

(female caregiver-6-E)

*“We have a credit association of about 23 women in our neighborhood. [The CBRW] asked us if there are mentally ill members in our families and advised us to take the ill people to the health center. The women also gave her information her about the [mentally ill people] in their houses or in their neighborhood. One of the women, for instance, told her that her brother is mentally ill. [The CBRW] told her to take him to the health center and he is showing significant improvement now.”*

(female caregiver-1-E)

*“[The man with schizophrenia] has a person helping him with food, there is another person who covers his expense for his medication…another person also gave him a place to stay. There are also people who are arranging to help him get a job. The CBRW has played a big role in arranging for …people to help him in every aspect and make him become successful and profitable.. a very huge role in helping them get free treatment. It is because of the CBRW’s role that these opportunities were created for him.”*

(male supervisor-E)

*I have made an effort, by getting close with them rather than discriminating them…ah… , by revitalizing their mind…ah…when you show them clear love they become mentally healthy. In the hope of achieving this, there was a time where I invited them to my home…I did this because I wanted him to have a free mind.”* (priest-5-E)

### Assumption 3c: Family support groups are perceived to be useful despite not having saving and loans element

*“[my husband] said he will not gather with other [mentally] ill people and refused to attend the meeting. But I told Mrs [..] that her daughter will recover if she takes her to the hospital and let her start taking the medication properly. The mother took her daughter to the health post and she is seeing significant improvement now”*

(female caregiver-1-E)

*“[the family support group] was good. It is good to get relief. I think it is helpful for us as it gives us relief from our daily suffering. ... It is even very helpful and refreshing as we meet. .... Is it not good to rest?”*

(woman with schizophrenia -11-E)

*“the (patient) was not willing on this. He feels bad. If he is told to meet with other individuals with the illness, he won’t be happy about it. He even was sometimes not happy about the home visit. If he is asked to go to such place and meet with other individuals, it wouldn’t be good.”*

(female caregiver-6-E)

*“There is one thing which is not good. She told to us who are mentally ill to meet at the centre of the kebele. However this is difficult to me. I: Eh… do you mean the family support group? P: Yes. I: Is it not comfortable to you? P: Yes. I: Why was that? P: If I go there for meeting, I could lose many things in my house. …eh… I feel as I am going to see other’s life…. Am I going to teach the group there? …eh… I don’t think that is comfortable to me.”*

(man with schizophrenia-1-E)
