# Supplementary material for: Community-based rehabilitation intervention for people with schizophrenia in Ethiopia (RISE): a 12 month mixed methods pilot study
Source: BMC Psychiatry. 2018 Aug 3;18:250. doi: 10.1186/s12888-018-1818-4 (PMC6091097; doi:10.1186/s12888-018-1818-4)
Supplement: Supplementary file 8 — Individiual level process and outcome data. Word document. (DOCX 17 kb) [file 12888_2018_1818_MOESM8_ESM.docx]

| **CBR participant ID** | **Months CBR received** | **Number home visits** | **Optional modules undertaken/ indicated modules (%)*** | **Optional goals achieved/selected goals (%) *** | **Meetings with community members** | **Referrals to health centre/ heath extension worker** |
| --- | --- | --- | --- | --- | --- | --- |
| **1** | 12 | 22 | 5/7 (71.4) | 8/11 (73) | 1 | 2 |
| **2 **** | 12 | 21 | 5/5 (100) | 4/9 (44) | 0 | 2 |
| **3 **** | 10*** | 17 | 5/5 (100) | 2/10 (20) | 0 | 1 |
| **4** | 8 | 19 | 4/5 (80) | 1/8 (13) | 3 | 2 |
| **5** | 12 | 17 | 5/5 (100) | 8/9 (89) | 10 | 1 |
| **6** | 11 | 19 | 4/4 (100) | 6/8 (75) | 0 | 0 |
| **7** | 12 | 24 | 7/7 (100) | 8/8 (100) | 5 | 1 |
| **8** | 11 | 27 | 7/7 (100) | 7/11 (64) | 4 | 4 |
| **9** | 12 | 23 | 4/5 (80) | 6/9 (67) | 1 | 1 |
| **10** | 12 | 22 | 6/7 (85.7) | 5/6 (83) | 0 | 2 |
| **Mean** | **11.2** | **21.1** | **5.4/5.7 (91.7)** | **5.5/8.9 (62)** | **2.4** | **1.6** |

**RISE pilot individual process data**

*All participants started all core modules and achieved all four core goals **Co-morbid intellectual disability*** Participant (ID 3) died

**RISE pilot individual outcome data**

| **ID** | **Clinical global impression (1=normal 7= most severely ill)** | | | **Depression (PHQ-9 total)** | | | **Alcohol use (AUDIT total)** | | | **Discrimination (total DISC score)** | | | **Caregiver burden (IEQ total)** | | |
| --- | --- | --- | --- | --- | --- | --- | --- | --- | --- | --- | --- | --- | --- | --- | --- |
|  | **0 m** | **6 m** | **12 m** | **0 m** | **6 m** | **12 m** | **0 m** | **6 m** | **12 m** | **0 m** | **6 m** | **12 m** | **0 m** | **6 m** | **12 m** |
| **1** | Missing | 2 | 4 | 2 | 3 | 9 | 7 | 15 | 10 | 2 | 0 | 0 | 35 | 48 | 59 |
| **2 *** | 7 | 5 | 5 | 11 | 9 | 2 | 0 | 2 | 0 | 0 | 3 | 0 | 69 | 56 | 47 |
| **3 *** | 4 | 7 | Participant died | 13 | 19 | Participant died | 0 | 0 | Participant died | 2 | 0 | Participant died | 43 | 21 | Participant died |
| **4** | 4 | 2 | 1 | 13 | 5 | 13 | 5 | 5 | 7 | 29 | 11 | 7 | 37 | 19 | 22 |
| **5** | 3 | 3 | 4 | 10 | 12 | 8 | 22 | 13 | 2 | 35 | 34 | 24 | Missing | 27 | 33 |
| **6** | 5 | 2 | Participant refused | 15 | 2 | Participant refused | 27 | 4 | Participant refused | 1 | 0 | Participant refused | 61 | 26 | Participant refused |
| **7** | 6 | 4 | 2 | 4 | 11 | 1 | 3 | 6 | 4 | 0 | 0 | 0 | 37 | 21 | 36 |
| **8** | 7 | 1 | 2 | 12 | 1 | 4 | 0 | 16 | 3 | 4 | 4 | 0 | 70 | 27 | 28 |
| **9** | 6 | 3 | 2 | 6 | 0 | 1 | 4 | 4 | 0 | 0 | 0 | 0 | 56 | 16 | 16 |
| **10** | 4 | 2 | 1 | 10 | 7 | 3 | 0 | 0 | 0 | 2 | 0 | 0 | 46 | 55 | 15 |
| **Median (IQR) or %** | **0% borderline /normal** | **50% borderline /normal** | **62.5% borderline /normal** | **10.5 (6,13)** | **6 (2,11)** | **3.5 (1.5,8.5)** | **3.5**  **(0,7)** | **4.5 (2,13)** | **2.5 (0,5.5)** | **2 (0,4)**  **70% any** | **0 (0,4)**  **40% any** | **0 (0,3.5)**  **25% any** | **46 (37,61)** | **26.5 (21,48)** | **30.5 (19,41.5)** |

* Co-morbid intellectual disability
